# A Model of Hormonal Regulation of Stamen Abortion during Pre-Meiosis of *Litsea cubeba*

**DOI:** 10.3390/genes11010048

**Published:** 2019-12-31

**Authors:** Zilong Xu, Yangdong Wang, Yicun Chen, Hengfu Yin, Liwen Wu, Yunxiao Zhao, Minyan Wang, Ming Gao

**Affiliations:** 1 State Key Laboratory of Tree Genetics and Breeding, Chinese Academy of Forestry, Beijing 100091, China; 18726089100@163.com (Z.X.); wangyangdong@caf.ac.cn (Y.W.); chenyc@caf.ac.cn (Y.C.); hfyin@sibs.ac.cn (H.Y.); wuliwenhappy@caf.ac.cn (L.W.); zyx_yunxiao@163.com (Y.Z.); w524270986@163.com (M.W.); 2 Research Institute of Subtropical Forestry, Chinese Academy of Forestry, Hangzhou 311400, China

**Keywords:** *Litsea cubeba*, sex differentiation, stamen abortion, plant hormone, salicylic acid

## Abstract

*Litsea cubeba* (Lour.) Pers., a popular essential oil plant, is a dioecious species with degenerative sexual organs in both male and female individuals. Yet, the mechanism of degenerative organs development in male and female flowers is poorly understood. Here, we analyzed the morphological characters of degenerative organ development by morphological and histological observations, and determined the critical stage of abortion that occurs at pre-meiosis in male and female flowers. We also conducted RNA sequencing (RNA-seq) to understand the genetic basis of stamen abortion in female flowers. The differentially expressed genes (DEGs) were identified during the staminode development in female flowers; functional enrichment analysis revealed some important biological pathways involved the regulation of stamen abortion, including plant hormone signal transduction, phenylpropanoid biosynthesis, flavonoid biosynthesis and monoterpenoid biosynthesis. Furthermore, 15 DEGs involved in the hormone pathways were found to regulate stamen development. By HPLC-MS/MS analysis, there were a salicylic acid (SA) content peak and the gibberellin (GA) content lowest point in the abortion processes in female flowers, suggesting a vital function of hormonal processes. Co-expression network analysis further identified several hub genes that potentially played significant roles in the stamen abortion of *L. cubeba*. Taken together, we proposed a model involved in plant hormones pathways underlying stamen abortion during pre-meiosis in female flowers of *L. cubeba*.

## 1. Introduction

Sex differentiation plays an important role in fruit set and seed production, and its regulation is controlled by genetic factors, hormones, as well as environmental conditions [1,2,3]. To explore male and female roles, unisexual flowers are typically used as starting material for investigating the mechanisms of sex differentiation and determination in plants. Historically, unisexual flowers are divided into two types based on the processes of morphogenesis and development [3]. The first type of flowers, which included those of *Vitis vinifera* subspec. *Sylvestris* [4], are hermaphroditic at the early stage of development, and then as the stamens (male floral organs) or pistils (female floral organs), degenerate or abort and they become floral reproductive organs without functions, the flowers become unisexual flowers. The second type of flowers, which include those of *Spinacia oleracea* [5], are a completely unisexual, that is, they do not contain any residual sex organs.

Among angiosperms, unisexual flowers are divided into monoecious and dioecious taxa, with about 6% being dioecy and 7% being monoecy [3]. Degenerative floral organs occur in four developmental stages, including Stage 0 (before sex organ primordium); Stage 1 (in the early development of sex organ primordium); Stage 2 (pre-meiosis) and Stage 3 (post-meiosis) [3]. Based on previous literature on the arrest of development among above the four stages (Table 1), degenerative mechanisms are known for Stages 0 and 1. By contrast, little is known about organ degeneration in Stage 2. Furthermore, only female flowers have been reported in Stage 2 and the mechanism of regulation has not yet been revealed.

*Litsea cubeba* (Lour.) Pers., belonging to the Lauraceae family, is native and widely distributed in the east and south of China and other areas of Southeast Asia [15]. It is considered to be a promising tree due its raw materials, which are used as cosmetics, Chinese traditional medicine, fragrance enhancers in foods, and aromatic oils [16,17]. Fruit is main raw material, therefore, our attention is on the development of staminodes in female flowers because they are associated with pollination efficiency [18]. Morphological and anatomical studies have revealed that both male and female flowers have degenerative organs in *L. cubeba* [19]. This provides a starting point for studying the molecular regulatory mechanisms that contribute to degradation during pre-meiosis. Furthermore, studying stamens abortion in *L. cubeba* is helpful for pollination efficiency or a better understanding of sex differentiation. To understand the molecular mechanisms underlying sex determination in *L. cubeba*, we performed morphological investigation, RNA-Seq analysis, and hormone detection in male and female flowers in *L. cubeba*. The present investigation provided valuable data related to regulatory hormone signaling factors that establish a foundation for future studies on sex determination of this important dioecious tree.

## 2. Materials and Methods

### 2.1. Plant Materials

**Figure 1:**
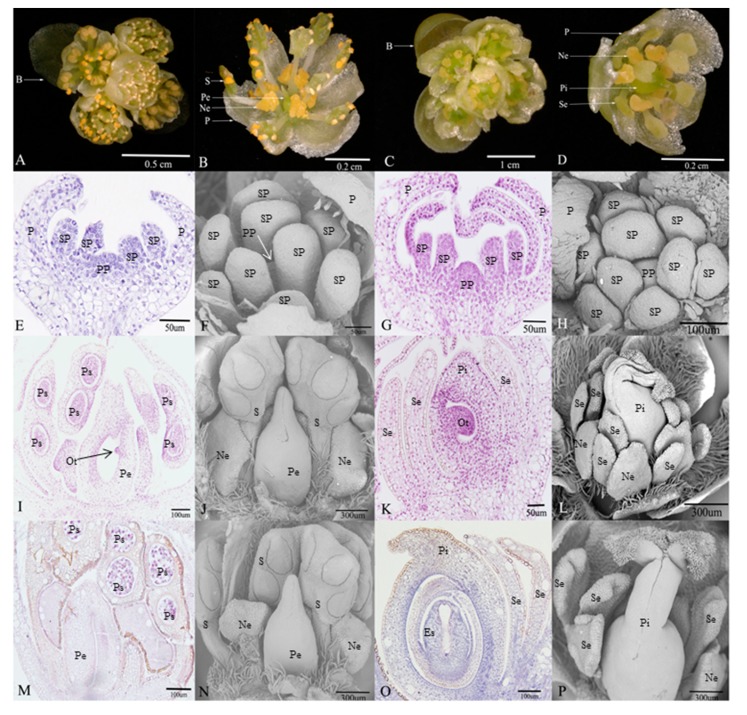
Morphological comparison of flower structure between male and female, and the microscopical features of degenerative organs in male and female flowers during the three developmental stages in *Litsea cubeba*. (**A**,**B**) Umbel inflorescences and floral organ structures in male flower; (**C**,**D**) umbel inflorescences and floral organ structures in female flower; (**E**,**F**) stamen primordium and pistil primordium occur in male flower; (**I**,**J**) the early stage of pistillode and stamen pollen sac formation in male flower; (**M**,**N**) pollen maturity stage, morphological structure of pistillode in male flower; (**G**,**H**) stamen primordium and pistil primordium occur in female flower; (**K**,**L**) the early stage of staminodes and embryo sac formation in female flower; (**O,P**) embryo sac mature stage, morphological structure of staminode in female flower. (B: bracts; S: stamens; Pe: pistillode; Ne: nectaries; P: perianth; Se: staminode; Pi: pistil; SP: stamen primordium; PP: pistil primordium; Ps: pollen sac; Es: embryo sac; Ot: ovule tissue).

Male and female floral buds of *L. cubeba* were collected in Fuyang’s Xinsha Island, Hangzhou, Zhejiang Province, China. Based on observations of morphological and anatomical features of flower development in *L. cubeba* [19,20], floral buds were randomly collected every 5 days between August and November, 2017. Floral buds were collected for morphological identification (fixed by formalin-acetic acid- alcohol (FAA) and 4% glutaraldehyde solution), meanwhile, the same development stage of samples used for RNA sequencing and determination of plant hormones were also separately frozen in liquid nitrogen and stored at −80 °C prior to analysis. The development stages of floral buds were identified firstly by morphological observations, and three representative degenerative developmental processes were selected as follows: sex organs primordium are both initiated in male and female floral buds at Stage 1 (M1: Figure 1E,F; F1: Figure 1G,H), pre-meiosis at Stage 2 (M2: Figure 1I,J; F2: Figure 1K,L), and post-meiosis at Stage 3 (M3: Figure 1M,N; F3: Figure 1O,P). Then RNA sequencing and determination of plant hormones were performed. In addition, three biological replicates of two sex types in the three representative developmental stages were collected for RNA sequencing and determination of plant hormones separately.

### 2.2. Microscopice and Histological Observations

To understand the detailed process of floral bud development and verify the differentiation period, female and male floral buds were paraffin sectioned based on the methods of Ye [21]. Fresh floral buds were first photographed under stereomicroscope (LEICA S8APO, Wetzlar, Germany), then fixed in a FAA fixative and evacuated, after which the FAA fixative was replaced once for at least 24 h. After that, the samples were dehydrated by concentration of 30%, 50%, 70%, 80%, 90%, and 100% ethanol and then treated with xylene, followed by embedding in paraffin blocks. The material was cut into thin paraplast sections (6–10 μm) using a rotary microtome (Thermo HM325, Waltham, MA, USA). The material was subsequently deparaffinized by xylene and ethanol, and then stained with hematoxylin and eosin solution. Finally, the slices were coated with neutral gum and imaged using an Olympus BX53 microscope (Olympus, Tokyo, Japan).

### 2.3. Scanning Electron Microscopy (SEM)

Floral buds from three developmental stages (M1/F1, M2/F2 and M3/F3) were fixed in 4% glutaraldehyde solution for more than 12 h and stored at 4 °C. The buds were then rinsed three times (2 h each) in 0.1 M phosphate buffered saline (PBS). Next, the samples were dehydrated for 15 min each with ethanol concentrations of 30%, 50%, 70%, 80%, 90%, and 100%. The buds were washed three times (30 min each) in tert-butanol, then dried with a CO_2_ critical point dryer (Quorum EMITECH-K850X, East Sussex, Britain) and metalized with gold-palladium. Imaging and analyzing were performed using a scanning electron microscope (PHENOM PRO, Eindhoven, The Netherlands).

### 2.4. Total RNA Extraction and Sequencing

Total RNA was extracted from male and female floral buds of *L. cubeba* using Trizol reagent (Invitrogen, CA, USA) following the manufacturer’s procedure and immediately frozen at −80 °C until use. Total RNA quantity and purity were respectively analyzed using a Bioanalyzer 2100 and an RNA 6000 Nano LabChip Kit (Agilent, CA, USA) with RIN number > 7.0. Approximately 10 µg of total RNA representing a specific adipose type was subjected to isolate Poly-A mRNA with poly-T oligo attached magnetic beads (Invitrogen). Following purification, the poly-A–or poly-A + RNA fractions were fragmented into small pieces using divalent cations under elevated temperature. Then the cleaved RNA fragments were reverse-transcribed to create the final cDNA library in accordance with the protocol for the mRNA-Seq sample preparation kit (Illumina, San Diego, USA). The average insert size for the paired-end libraries was 300 bp (±50 bp). Finally, we performed paired-end sequencing on an Illumina Hiseq 4000 at the (lc-bio, China) following the vendor’s recommended protocol.

### 2.5. De Novo Assembly, Unigene Annotation and Functional Classification

Firstly, cut adapt and in-house Perl scripts were used to remove the reads that contained adaptor contamination, low quality bases, and undetermined bases [22]. Then, sequence quality was verified using FastQC, including the Q20, Q30, and GC-content of the clean data. All downstream analyses were based on clean, high quality data. De novo assembly of the transcriptome was performed with Trinity 2.4.0 [23]. Trinity groups transcripts into clusters based on shared sequence content. Such transcript clusters are loosely referred to as “genes.” The longest transcript in the cluster was chosen as the “unigene” sequence (aka unigene). All assembled unigenes were aligned against the non-redundant (NR) protein database (http://www.ncbi.nlm.nih.gov/), gene ontology (GO) (http://www.geneontology.org), SwissProt (http://www.expasy.ch/sprot/), Kyoto Encyclopedia of Genes and Genomes (KEGG), (http://www.genome.jp/kegg/), and eggNOG (http://eggnogdb.embl.de/) databases using DIAMOND with a threshold of E value < 0.00001 [24]. The clean data sets are available at the NCBI SRA with the accession number: SRR10053824, SRR10053795, SRR10053793, SRR10053782, SRR10053780, SRR10053770, SRR10053769, SRR10053767, SRR10053765, SRR10053109, SRR10052556, SRR10052491, SRR10052460, SRR10052459, SRR10052050, SRR10052049, SRR10051549, and SRR10051547.

### 2.6. Differentially Expressed Gene (DEG) Analysis

Salmon was used to assess expression level for unigenes by calculating transcripts per million (TPM) [25,26]. The differentially expressed unigenes were selected with |log_2_ (fold change)| > 1 with statistical significance (FDR (false discovery rate) value < 0.01) by R package edge R [27]. Next, GO and KEGG enrichment analyses were performed on the differentially expressed unigenes by Perl scripts in house.

### 2.7. Network Analysis of SA Biosynthesis and Signaling Pathway

To identify key hub genes in SA pathway significantly connected with other related genes, we conducted the weighted gene co-expression modules using an R package for weighted gene co-expression network analysis (WGCNA) with a TPM value > 3.0 [28]. The co-expressed gene network of hub genes and their corresponding connective genes were constructed using Cytoscape software [29].

### 2.8. qRT-PCR Verification of Transcriptome

Twelve DEGs were selected for qRT-PCR analysis using the RNA samples from three developmental stages of female and male flowers, which were used to verify the validity of transcriptome data. RNA isolation was performed using the RNeasy Plant Mini Kit (Qiagen, www.qiagen.com) according to the supplier’s instructions. First strand cDNA synthesis was performed using PrimeScript™ 1st Strand cDNA Synthesis Kit (Takara, www.takarabiomed.com) according to the supplier’s protocol using oligo (dT) primer. Quantitative real-time RT-PCR was performed using TB Green^®^ Premix Ex Taq™ II (Tli RNaseH Plus) from TaKaRa (China) and experimental reaction conditions as followed: 30 s for denaturation at 95 °C, followed by 40 cycles of 5 s for denaturation at 95 °C, 20 s for annealing at 60 °C, and 20 s for extension at 72 °C. Gene-specific primer design was based on transcriptome sequencing results using Primer 5 (Table S1). The ubiquitin-conjugating enzyme E2 (UBC) gene was used as an internal reference gene for PCR analysis [15]. The relative expression levels of the selected DEGs were measured using the 2^−^^ΔΔCt^ method [20].

### 2.9. The Detection of Phytohormone Levels

Samples were analyzed for 1-aminocyclopropane-1-carboxylicacid (ACC) content (ACC is ethylene precursor) by high-performance liquid chromatography tandam mass spectrometry (HPLC-MS/MS) and internal ACC standards were purchased by the Company of BaiLin Wei (China). Firstly, samples were accurately weighed to ~0.6 g and ground to a powder in liquid nitrogen. Five ml of deionized water was added to the powder, sonicated in a water bath for 30 min, and then place at 4 °C. After centrifuge at 10,000 r/min for 5 min, we extracted the supernatant, adjusted the value of pH to 4.0, added 20 mL of chloroform, mixed by shaking, and then centrifuged at 10,000 r/min for another 5 min. The supernatant was next filtered through a MCX column and activated by 3 mL of methanol and 3 mL of deionized water. The MCX column was rinsed with 2 mL of methanol and 1 mL of deionized water. The column was eluted with 5 mL of 1 M ammonia water and passed through a 0.22 μm filter for HPLC-MS/MS. The samples were tested by UPLC (Waters ACQUITY UPLC Xevo TQ) and used electrospray ionization (ESI) as the ion source for multi-channel detection mode scanning. The data of ACC was obtained using monitoring conditions for protonated plant hormones ([M+H]^+^) (Table S5)

After sampling, the levels of indole acetic acid (IAA), gibberellin 3 (GA3), gibberellin 1 (GA1), gibberellin 4 (GA4), gibberellin 7 (GA7), trans-zeatin nucleoside (TZR), salicylic acid (SA), jasmonic acid (JA), methyl salicylate (MESA), and methyl jasmonate (MEJA) were tested, and internal standards, including IAA, GA1, GA3, GA4, GA7, TZR, SA, JA, MESA and MEJA, were purchased from Sigma Company (USA). Samples were collected by isopropanol/water/hydrochloric acid extraction. The samples were ground to a powder in liquid nitrogen and accurately weighed to ~1.5 g. The powder was added to 10 mL of isopropanol/hydrochloric acid extraction buffer and shaken for 30 min at 4 °C. An amount of 20 mL dichloromethane was added and the samples were further shaken for 30 min and centrifuged at 13,000 r/min for 5 min to extract the lower organic solution. Next, the organic solution was dried with nitrogen in the dark, and dissolved in 400 μL methanol (0.1% formic acid). Finally, the sample was passed through a 0.22 μm filter and tested by HPLC-MS/MS. Analysis was done by HPLC (Aglient 1290, USA) coupled to a triple-stage quadrupole mass spectrometer (SCIEX-6500Qtrap (MSMS), USA) equipped with an ESI interface. The date of IAA, GA3, GA1, GA4, GA7, TZR, SA, JA, MESA, and MEJA were obtained based on selected reaction monitoring conditions for protonated or deprotonated plant hormones ([M+H]^+^ or [M–H]^−^) (Table S5).

### 2.10. Statistical Analysis

The data were analyzed using IBM SPSS Statistics 23 software (SPSS Inc., Chicago, IL USA). One-way ANOVA was performed and Duncan multiple-comparison test was used to detect the differences between the means. A *p* value < 0.05 was considered significant. The average levels of three biological replicates were shown in all figures. The data were described as mean values ± standard deviation.

## 3. Results

### 3.1. Degenerative Organ Formation of Female and Male Flowers in L. cubeba

Flowers of *L. cubeba* contain obvious umbel inflorescences and four bracts. In female flowers (Figure 1C), each inflorescence has five pistillate flowers. Each female flower consists of a six-lobed perianth and nine staminodes that lack biological functions. Meanwhile, irregular nectaries are born at the base of the inner staminodes. A pistil surrounded by staminodes (Figure 1D). There are similar floral structures, such as perianths and nectaries, in male flowers (Figure 1A), but the most obvious difference is that the stamens and pistillode lack stigma, and styles are shortened or missing (Figure 1B).

In the early development of male and female floral buds in *L. cubeba*, male and female sex organs primordium are both initiated in developing buds (Figure 1E–H). During male flower development, the stamens that originated from stamen primordium normally develop into pollen sacs (Figure 1I) and undergo the process of meiosis, eventually forming functional stamens. However, with the development of the pistil primordium, pistil development arrests in the ovule tissue before meiosis, and the pistil has no stigma and a short style (Figure 1J). Until pollen matures, the structure and size of pistil remain unchanged (Figure 1J,N). In the development of the female flower, during female flower development, we observed normal pistils with stigma, styles, ovary structure, and staminodes (Figure 1L,P). We found that ovule tissue formed (Figure 1K) and underwent meiosis resulting in an embryo sac structure (Figure 1O). In contrast, we failed to observe sporogenous cells and pollen sac formation (Figure 1K,O).

### 3.2. De Novo Assembly of the Transcriptome and Functional Annotation

Libraries were constructed on M1–M3 and F1–F3 samples and Illumina Sequencing was performed, and each library was subjected to three biological replicates to obtain a total of 133.82 Gbp of raw transcript data. Effective clean transcript data of 127.07 Gbp were obtained through filtration and impurity removal (removing sequencing linker sequences, repeated redundant sequences, poor quality sequences, etc.). Clean reads accounted for more than 97%, Q20 and Q30 were more than 97%, 92%, and GC content accounted for more than 46% (Table S2–1). Next, all samples were mixed and assembled, which were finally normalized to obtain Unigene. A total of 103,921 Unigenes were obtained with the GC content occupying 42.3%, the average length was 428 bp, and the N50 value was 1412 bp (Table S2–2). It indicates that the coverage and saturation of the samples are good and can be used for the further experimental analysis. For the assembled unigenes, we used the new alignment software DIAMOND and blastx to add corresponding functional annotations [24]. Based on the high conservation of similar functional genes in sequence (nucleic acid sequence or protein sequence) among different species, we selected six authoritative databases, namely NR, GO, KEGG, Pfam, Swissprot and COG (Cluster of Orthologous Groups of proteins), with annotation rates of 39.40%, 29.38%, 19.69%, 29.99%, 30.31% and 7.33%, respectively (Figure S1). Species distribution analysis showed that unigenes of *L. cubeba* matched those of various plant species (Figure S1), such as *Nelumbo_nucifera* (24.4%), followed by *Oryza_sativa* (10.13%), and *Vitis_vinifera* (8.3%).

Without considering the different group comparison information, we performed an overall statistical analysis on the expression of genes in all samples. The relationship of transcriptome samples from the stages LC_M1/F1 to LC_M3/F3 were assessed by the Pearson correlation coefficient. It was found that the third biological replicate correlation in the F2 period was low (Figure S2). Similarly, the same result was found between samples by boxplot (Figure S2), therefore, the related unigene expression data and subsequent differential gene analysis were excluded.

### 3.3. Enrichment Analysis of DEGs in Female and Male Flower

To identify differentially expressed genes involved in abortion in female flower development, the unigenes were assessed in male and female flowers based on the aforementioned conditions. As a result, digital expression profiling showed that 16,635 genes were differentially expressed during the development of male flowers, while 10,524 genes showed differential expression during female flower development.

**Figure 2:**
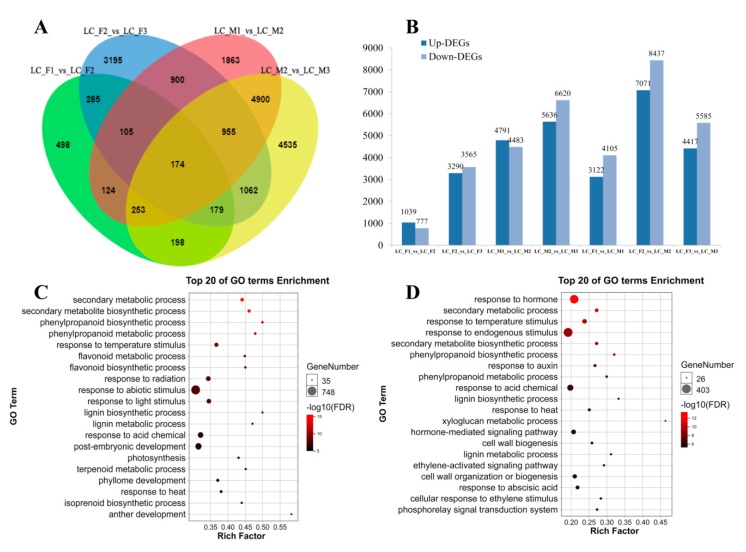
Analysis of RNA-seq in male and female flower. (**A**) Venn diagrams of differentially expressed genes (DEGs) among the three stages in the female and male flower; (**B**) up-DEGs and down-DEGs were showed in male and female flower; (**C**,**D**) top 20 of gene ontology (GO) terms enrichment in the male and female flower, respectively.

We identified 15,248 DEGs in male flowers (LC_M1_vs_LC_M2, LC_M2_vs_LC_M3) and 7928 DEGs in female flowers (LC_F1_vs_LC_F2, LC_F2_vs_LC_F3) (Figure 2A). Next, GO enrichment analysis was applied to the 15,248 DEGs and 7928 DEGs, and up-DEGs and down-DEGs were showed in Figure 2B, we found that up- and down-DEGs in male flower were more than that of female flower, and there was a clear upward trend followed by a downward tendency, and it peaks in the second specific developmental stage in the female flowers and male flowers. Then, the top 20 GO terms were presented for male flowers (Figure 2C) and female flowers (Figure 2D). In the male flower, the GO terms, such as secondary metabolic and biosynthetic process, were obviously enriched. In contrast, the GO terms were enriched in hormones, which indicated that hormones were involved in the development of female flowers. Furthermore, 15,248 DEGs in male flowers were also analyzed using KEGG pathway enrichment and the pathways of 5224 DEGs were annotated. The top ten KEGG pathways (Q-value < 0.01) with high representation of the DEGs in male flowers are shown (Table 2). Similar, 7928 DEGs in female flowers were subjected to KEGG pathway enrichment analysis, the 2815 DEGs with pathway annotation were performed, from which we identified the top seven KEGG pathways (Q-value < 0.01) with high representation of the DEGs. Plant hormone signal transduction (ko04075) pathways in male and female were markedly enriched with higher correlation in female flower.

Furthermore, in order to compare with the specific developmental stage in the female flowers and male flowers, we also identified unique 1160 DEGs of female and unmatched 8618 DEGs of male in FM12 (LC_M1_vs_LC_M2, LC_F1_vs_LC_F2, Figure 2A), and unmatched 4485 DEGs of female and sole 9886 DEGs of male in FM23 (LC_M2_vs_LC_M3, LC_F2_vs_LC_F3, Figure 2A). In FM12, the KEGG pathway enrichment analysis (Q-value < 0.01) that plant hormone signal transduction (ko04075) as important pathways, which demonstrated that plant hormone might be related to abortion of stamens in female flowers. In FM23, 4485 DEGs of female and 9886 DEGs of male were subjected to similar KEGG pathway enrichment analysis (Q-value < 0.01), and KEGG pathways with high representation in the DEGs were showed in Table 2. Furthermore, we analyzed the two stages (LC_M1_vs_LC_F1, and LC_M2_vs_LC_F2) of male and female flower development respectively. The GO and KEGG pathways enrichment analysis were performed (Figure S3). In the first stage (LC_M1_vs_LC_F1), the GO terms, such as salicylic acid, ethylene and other hormones, and plant hormone signal transduction as well as phenylpropanoid biosynthesis in the pathways enrichment were obviously enriched (Figure S3A,B). Similarly, the plant hormone and phenylpropanoid biosynthesis were also found in the second stage of the 20 top GO terms and KEGG pathways (LC_M2_vs_LC_F2) (Figure S3C,D). The expression trend of 459 DEGs related to plant hormone signal transduction in Table 2 were analyzed by short time-series expression miner software (STEM) in male and female flowers that should be clustered into eight profiles separately (Figure S4). In male flowers, 10 DEGs were down-regulated in Profile 0 that were related to IAA, ETH, BR and JA, and 17 DEGs were up-regulated in Profile 7 that were involved in IAA, JA, SA, etc. (Figure S4, Table S7). Similarly, 57 DEGs showed a decreased trend in Profile 0 and 26 DEGs displayed a opposed tendency in Profile 7 in female flower, which were associated with IAA, cytokinin (CTK), GA, etc. (Figure S4, Table S7).

### 3.4. Phytohormone Levels in Male and Female Flowers of L. cubeba

In order to explore whether the arrest of male and female flowers is regulated by hormones, the endogenous levels of IAA, gibberellins (GAs), jasmonic acids (JAs), cytokinin (CTK), ACC, and salicylic acids (SAs) in *L. cubeba* flowers at different stages were measured (Table 3). The level of IAA showed a decreasing trend with the highest value in M1 (3.67 ng/g) in males, while the content of F2 (1.94 ng/g) was higher than other stages in females (Table 3). The level of ACC in males was significantly higher in M2 (86.54 ng/g) than that of F2 (42.65 ng/g) in females, and a similar tendency was showed for both male and female flower developmental stages (Table 3). The CTK level of female flowers was an upward tendency with the highest level in F3 (1.19 ng/g), and the CTK content of male flowers dropped slightly to the lowest in M2 (0.33 ng/g) (Table 3). The content of JA displayed distinct expression patterns that male flowers reached the lowest level in the second stage (0.33 ng/g), while female flowers got the highest (1.67 ng/g), and MEJA content also showed different mode that there was a downward trend in female flowers and male flowers had the lowest content in the second stage (1.27 ng/g) (Table 3). For GAs, the content of GA3, and GA4 showed a similar trend with the highest value in M2 separately, while females displayed differential trend that GA3 level was highest in F1(3.59 ng/g) and GA4 level was lowest in F2 (0.06 ng/g). GA1 and GA7 levels were not measured at all stages. SA content was obviously higher than MESA content in male and female flowers. MESA content showed no difference. SA content was significantly increased from Stage F1 to Stage F2 and reached the peak (11.05 ng/g), then significantly decreased from Stage F2 to Stage F3 (Table 3).

### 3.5. Candidate Genes Involved in Stamen Developmentin L. cubeba

**Figure 3:**
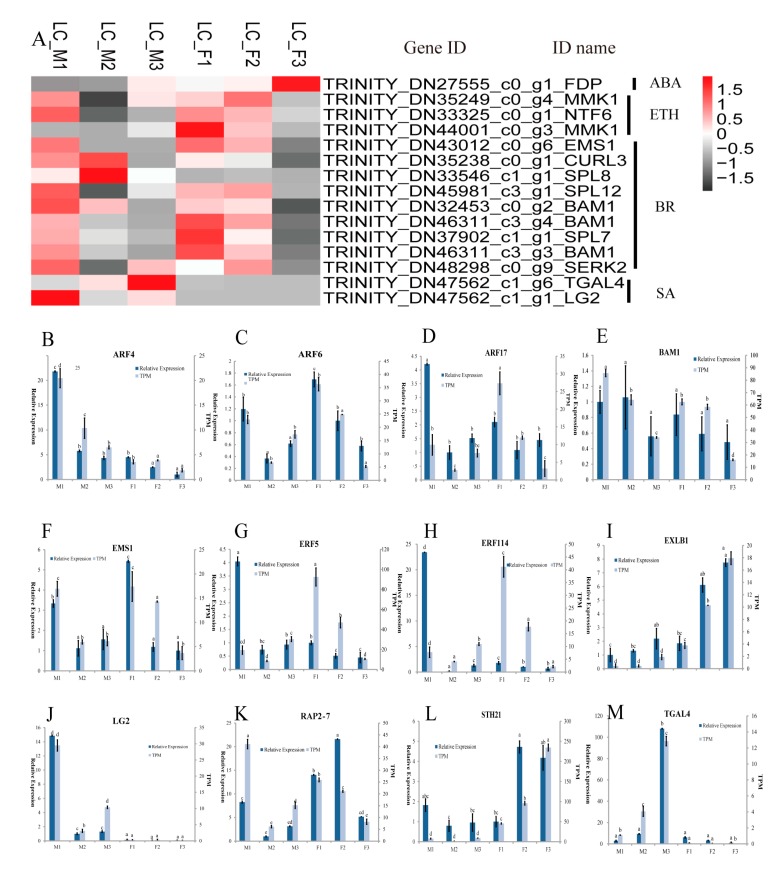
The expression pattern of candidate genes involved in the floral development in *L. cubeba*. (**A**) Heatmap analysis of key stamen developmental genes in male and female flower; (**B**–**M**) expression levels of *ARF4*, *ARF6*, *ARF17*, *BAM1*, *EMS1*, *ERF5*, *ERF114*, *EXLB1*, *LG2*, *RAP2-7*, *STH21* and *TGAL4* based on transcripts per million (TPM) and qRT-PCR data. Different letters represent significant difference in mRNA levels (*p* < 0.05). Error bars mean SD.

The KEGG enrichment analysis (Table 2) indicated that 459 DEGs were involved in plant hormone signal transduction pathways, in which 89 DEGs were from IAA, 52 DEGs from gibberellin (GA), 45 DEGs from abscisic acid (ABA), 146 DEGs from brassinosteroid (BR), 26 DEGs from cytokinin (CTK), 24 DEGs from jasmonic acid (JA), 56 DEGs from ethylene (ETH), and 21 DEGs from salicylic acid (SA) pathways (Table S6). These 459 DEGs were enriched in the GO terms related to stamen development: anther development (GO: 0048653), androecium development (GO: 0048466) and stamen development (GO: 0048443), etc. (Table S3), which included 15 DEGs. Furthermore, the selected15 DEGs related to stamen development exhibited differential expression patterns at different stages and were classified in SA, ETH, and BR (Figure 3A). Most of the 15 DEGs were highly expressed in the first two stages in the female or male flowers. Interestingly, the three genes (*SPL8*, *TGAL4*, and *LG2*) displayed three different expression patterns in male flowers, with relatively low expression levels in their female counterparts, in contrast, the MMK1 genes showed a decreased pattern in the female flowers, while there was relatively low in the level of RNA-seq expression in male flowers. The qRT-PCR analysis was applied to validate the expression of six genes related to floral stamen development and to confirm the validity of the transcriptome data (Figure 3B–M).

### 3.6. Co-Expression Networks Involved in the Staminodes of Female Flowers

**Figure 4:**
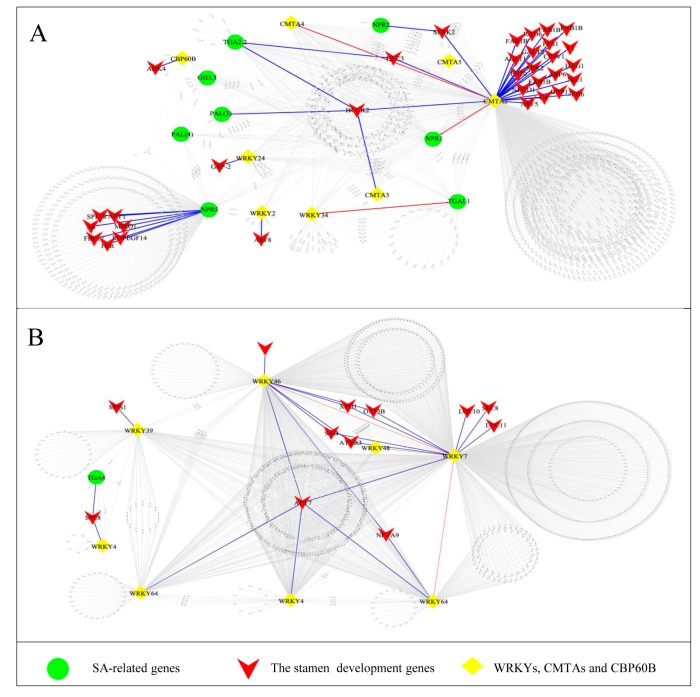
Co-expression networks analysis of genes in salicylic acid (SA) of female flowers. (**A**,**B**) Co-expression networks analysis of stamen development genes and SA-related genes.

To obtain the further information for the regulatory network of candidate genes (before meiosis) involved in stamen abortion in female flower in *L. cubeba*, we conducted a WGCNA analysis with TPM values > 3 for the 11 co-expression modules. Therefore, we chose and displayed a new co-expression network of SA-related genes that was built with the value of weight > 0.63 (Figure 4). The co-expression networks demonstrated that 2538 genes were divided into five parts. The two parts, including 1385 genes (Figure 4A) and 1055 genes (Figure 4B), contained the greatest number of genes. A number of stamen development genes were identified by GO enrichment, such as *HULK2*, *HAG1*, *ARF5*, *HEC3*, *ARF6*, *ARF8*, *APY7*, etc. (Figure 4A,B and Table S4). The SA-related genes of *NPR* (1,2,5), *PAL* (3,4), *TGA* (2.2,4,L1), *WRKY* (2,4,7,24,34,39,46,48,64,69), *CMTA* (2,3,4,5), *GH3.3* and *CBP60B* were identified in the network (Figure 4A,B). Furthermore, *CMTAs* (23 edges), *WRKYs* (13 edges) and *NPRs* (7 edges) were identified that had high connectivity with the stamen development genes. Interestingly, *WRKYs* had the highest connectivity in the SA biosynthesis and signaling pathways, suggesting they may have critical biological functions in regulating the abortion in the female flowers in *L. cubeba* (Figure 4A,B).

## 4. Discussion

### 4.1. L. cubeba as a Model for Stamens Abortion during Pre-Meiosis in L. cubeba

A previous study showed that the arrest of sex organ development can be occurred in different developmental stages in flowering plants. There are four stages of degenerative development: There is no opposite sex organ primordium in unisexual flower, such as *Spinacia oleracea* [5], which is called Stage 0 (before the inception of the stamens or carpel primordium); the unisexual flowers show little trace of opposite sex organ, like *Cucumis melo* [6,8], which is named Stage 1 (in the early stage of stamens or pistils); The degradation of stamens or pistil occurred, but no embryo sac or pollen sacs in Stage 2 (pre-meiosis of microspore or megaspore mother cells), for instance, residual stamens in female flowers generally showed no pollen sacs in *Diospyros lotus* [6,11]. Furthermore, the producing barren gametes in female flowers or sterile female gametophytes in male flowers was a characteristic in Stage 3 (post-meiosis), for example, anther development ceased before pollen occurred of female in *Asparagus officinalis* [12]. Cytological observation confirmed that pistil and stamen primordium appeared together in the early stage of pistil or stamen development, but the abortion of pistils or stamens fail to form embryo sac or pollen sacs in *L. cubeba*, which demonstrated that the abortion of stamens or pistil took place in Stage 2. In *Silene latifolia*, the arrested stamens of female flowers had no pollen mother cell [6,30]. In addition, there are no reports of pistil arrest in Stage 2, but there are still relevant findings in other stages, such as Stage 1 and 3 (Table 1). This result also suggested that the formation of female flowers is due to the degradation of stamens, and that male flowers are formed because of the arrest of pistil. Based on existing literature reports (Table 1), *L. cubeba* provides a suitable model for mechanistic exploration of pre-meiotic abortion.

### 4.2. Plant Hormones are Involved in Develpmental Processof Female and Male Flowers in L. cubeba

It has been reported that sex differentiation in plant was mainly determined by many factors, including sex chromosome, plant hormones, epigenetic modification and environmental factors [6,31,32]. Plant hormones affected floral organ abortion and sex formation, and evidence has uncovered many sex-determining genes and pathways related to plant hormones. In *Cucumis melo*, three sex-determining genes, which included *CmWIP1*, *CmACS11* and *CmACS7*, were identified and established a regulation model associated with the ETH synthesis [8,9,10]. In maize, JA plays a key role in the development of stamens, and *Tasselseed 1* mutants in the JA pathway had only female flowers [33]. CTK also affects sex determination, such as in *Kiwifruit* (*Actinidia* spp.), and gynoecia development was partially restored by the application of exogenous CTK in male flowers [2]. RNA-seq analysis in male and female indicated that phenylpropanoid biosynthesis, flavonoid biosynthesis and hormone signaling transduction were obviously enriched in male flowers, which demonstrated plant hormones, such as GA and SA, would be related to development process in male flowers. Similarly, the hormone signaling transduction pathway was also significantly enriched in the female flowers, which indicated that the plant hormone would be involved in development process of female flowers. Furthermore, RNA-seq analysis was performed in the specific developmental stage, such as F12 (F1,160) and LC_M1_vs_LC_F1. SA, ETH and other hormones were obviously enriched in female flowers, and the abortion of stamens in the female flowers in *L. cubeba* would be regulated by plant hormones. The detection of phytohormone levels showed that ACC, SA, JA and MEJA had significant differences, and sex differentiation and formation were affected by these hormones [3,34,35,36]. So these hormones would play a vital function in degenerative development of female and male flowers. We identified 15 hormone-related genes that might be involved in abortion of stamen of female flower development, such as *EMS1*, *TGAL4*, and *LG2*.The *EMS1* gene played a key function on anther cell differentiation of the early stamens development, and the two (*TGAL4*, *LG2*) genes affected early anther lobe development [37,38,39]. Thus, the expression of hormone-related genes might regulate the stamens abortion of female flower in *L. cubeba*.

### 4.3. GA or SA Mediates the Degeneration of Stamens of Female in L. cubeba

We found that terpenoid biosynthesis and metabolic, plant hormone signal transduction, fatty acid metabolism and phenylpropanoid biosynthesis were enriched in Table 2, and compared with the first/second stage (LC_M1_vs_LC_F1, and LC_M2_vs_LC_F2) in male and female flowers, SA response, ETH stimulus, fatty acid metabolism and phenylpropanoid biosynthesis were one of the significant enrichment (Figure S3). Terpenoid biosynthesis and metabolic were associated with GA pathway [40]. Phenylpropanoid and fatty acid could provide important precursors for SA and JA/MEJA biosynthesis respectively [41,42]. In *L. cubeba*, plant hormones content was measured by HPLC-MS/MS, which demonstrated that GA3/GA4 and MEJA contents were decreased from LC_F1 to LC_F2. The level of SA, JA and ACC were the highest expressed in LC_F2 during female floral development. GA and JA were considered to promote stamen development, such as the application of exogenous GA promoted male flower expression in *Rumex acetosa* [43] and the male development was inhibited by GA- or JA-deficient mutant [42,44]. The abortion of stamens would be promoted by reduced GA or JA content in *L. cubeba*, but JA content showed an increased trend from LC_F1 to LC_F2 in female. ACC was considered to mainly promote female flower formation. For example, the stamens were inhibited by high level of ACC in melon and cucumber [33]. However, compared with female flowers, the level of MEJA and ACC showed a similar trend from LC_M1 to LC_M2 in male flowers, so abortion of stamens would not be influenced by MEJA or ACC in *L. cubeba*. It was reported that flowering is delayed in SA-deficient mutants of *Arabidopsis thaliana* [41], and the early flowering phenotype was formed in the *AtSIZ1* mutant of *Arabidopsis thaliana*, which was dependent on elevated SA levels [45]. In rice, it was suggested that floret fertility and pollen viability were significant reduced by high level of SA [34]. In *L. cubeba*, the formation of stamens might be suppressed by the SA accumulation in early development in female flower. Due to SA, it was widely enriched in the development of female and male flowers, including the specific comparison of male and female development stages. Furthermore, co-expression networks analysis indicated *PALs*, *TGAs* and *NPRs* showed high connection with the stamen development genes, such as *HULK2*, *HAG1*, etc. *CMTAs* and *WRKYs* were the vital bridge between them. *PALs* that encode enzymes serves as a critical regulator in SA biosynthesis and *TGAs* and *NPRs* were regulators of SA-mediated transcriptional signaling [41,46]. It was reported that the member of *CMTAs* and *WRKYs* regulated SA accumulation [46,47]. Thus, the development and formation of staminodes could be regulated by the *CMTAs* and *WRKYs* in SA biosynthesis. GA or SA might play key roles on abortion of stamens of female flowers in *L. cubeba*.

**Figure 5:**
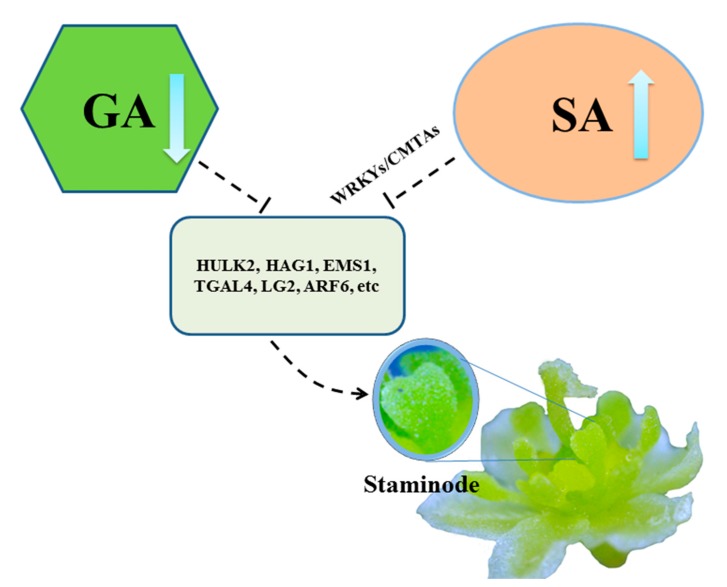
Hypothetical model for stamens abortion of female flowers in *L. cubeba*. The proposed pathway involved in SA biosynthesis (organ oval) or gibberellin (GA) biosynthesis (green polygon), then the normal expression of stamens developmental genes such as *HULK2*, *HAG1*, *EMS1*, *TGAL4*, *LG2* and *ARF6* (dark green square), resulting in the formation of staminodes.

Additionally, we constructed a hypothetical model for abortion of female flowers in *L. cubeba* (Figure 5). With increase of SA biosynthesis or decrease of GA biosynthesis in the early development (before pre-meiosis) in the female flower in *L. cubeba*, the normal expression of stamens genes such as *HULK2*, *HAG1*, *EMS1*, *TGAL4*, and *LG2*, etc., resulting in the formation of staminodes. Thus, stamens abortion occurred in the female flower.

## 5. Conclusions

We conclude that the abortion of male and female flowers occurred during pre-meiosis in *L. cubeba* by cytological observation. The floral organ development of males and females would be involved in plant hormones. Furthermore, the arrest of stamens in female flowers would be related to plant hormones, especially SA or GA biosynthesis, which might regulate the formation of staminodes. We constructed a hypothetical model of abortion of stamens in female flower in *L. cubeba*. This study provides a valuable foundation for understanding pre-meiotic stamen abortion in female flowers.

## Figures and Tables

**Table 1:** Stages, species and regulatory patterns in unisexual flowers in literature.

Degraded Stages	Species		Regulatory Patterns	Reference
	Male	Female		
Stage 0 (before sex organs primordium)	NA	*Spinacia oleracea* *Thalictrum dioicum* *Quercus suber* *Populus tomentosa*	Male flowers are regulated by B-class gene in *Spinacia oleracea*.	[5,6,7]
Stage 1 (in the early development of sex organs primordium)	*Cucumis melo* *Cucumis sativus* *Zea mays*	*Cucumis sativus* *Cucumis melo* *Kiwifruit*	Regulation of ethylene synthesis pathway and DNA methylation in *Cucumis melo*; The *SyGI* gene inhibits carpel development in *Kiwifruit*.	[2,6,8,9,10]
Stage 2 (pre-meiosis)	NA	*Asparagus officinalis* *Silene latifolia* *Diospyros lotus*	NA	[6]
Stage 3 (post-meiosis)	*Diospyros lotus* *Vitis vinifera* *Kiwifruit*	*Asparagus officinalis* *Vitis vinifera* *Vernicia fordii*	Epigenetic regulation (sRNA) in *Diospyros lotus*; The two-mutation model in *Asparagus officinalis*; the *FrBy* acts for the maintenance of male functions in *Kiwifruit*	[6,11,12,13,14]

NA: Not Applicable.

**Table 2:** The enrichment analysis on Kyoto Encyclopedia of Genes and Genomes (KEGG) pathway of DEGs among the three stages and the specific developmental stage (Q-value < 0.01).

Sample	Pathway ID	Pathway	Q-Value
MM (15,248)	ko01100	Metabolic pathways	0.000000
	ko00500	Starch and sucrose metabolism	0.000000
	ko01110	Biosynthesis of secondary metabolites	0.000000
	ko00940	Phenylpropanoid biosynthesis	0.000000
	ko04712	Circadian rhythm-plant	0.000001
	ko00941	Flavonoid biosynthesis	0.000002
	ko04075	Plant hormone signal transduction	0.000066
	ko00860	Porphyrin and chlorophyll metabolism	0.000820
	ko00942	Anthocyanin biosynthesis	0.006066
	ko00945	Stilbenoid, diarylheptanoid and gingerol biosynthesis	0.006066
FF (7928)	ko04075	Plant hormone signal transduction	0.000000
	ko00940	Phenylpropanoid biosynthesis	0.000003
	ko00941	Flavonoid biosynthesis	0.000005
	ko03010	Ribosome	0.000666
	ko03440	Homologous recombination	0.008624
	ko00902	Monoterpenoid biosynthesis	0.008624
	ko00073	Cutin, suberine and wax biosynthesis	0.009764
FM12 (F1,160)	ko04075	Plant hormone signal transduction	0.000000
	ko00941	Flavonoid biosynthesis	0.000004
	ko00514	Other types of O-glycan biosynthesis	0.000086
FM12 (M8,616)	ko01100	Metabolic pathways	0.000000
	ko00500	Starch and sucrose metabolism	0.000082
	ko00941	Flavonoid biosynthesis	0.000669
	ko00940	Phenylpropanoid biosynthesis	0.000669
	ko01110	Biosynthesis of secondary metabolites	0.001530
	ko00195	Photosynthesis	0.005490
	ko00860	Porphyrin and chlorophyll metabolism	0.008780
FM23 (F4,485)	ko03010	Ribosome	0.000000
	ko03440	Homologous recombination	0.005823
FM23 (M9,886)	ko00500	Starch and sucrose metabolism	0.000000
	ko01100	Metabolic pathways	0.000000
	ko00195	Photosynthesis	0.000779
	ko04712	Circadian rhythm-plant	0.001618
	ko00051	Fructose and mannose metabolism	0.004187
	ko00910	Nitrogen metabolism	0.005636
	ko01212	Fatty acid metabolism	0.005636
	ko00030	Pentose phosphate pathway	0.009996

The meaning of the abbreviation is as follows: MM (15,248): 15,248 DEGs in male flowers (LC_M1_vs_LC_M2, LC_M2_vs_LC_M3); FF (7928): 7928 DEGs in female flowers (LC_F1_vs_LC_F2, LC_F2_vs_LC_F3); FM12 (F1,160) and FM12 (M8,616): unique 1160 DEGs of female and unmatched 8616 DEGs of male in FM12 (LC_M1_vs_LC_M2, LC_F1_vs_LC_F2); FM23 (F4,485) and FM23 (M9,886): unmatched 4485 DEGs of female and sole 9886 DEGs of male in FM23 (LC_M2_vs_LC_M3, LC_F2_vs_LC_F3).

**Table 3:** The hormone levels in male and female flowers.

Hormones		Male Flowers Stages			Female Flowers Stages		
		M1	M2	M3	F1	F2	F3
IAA	IAA	3.67 ± 0.09a	2.35 ± 0.06b	1.28 ± 0.06e	1.68 ± 0.04d	1.94 ± 0.03c	1.24 ± 0.06e
SAs	SA	4.20 ± 0.16c	3.12 ± 0.04d	1.15 ± 0.05e	5.02 ± 0.20b	11.05 ± 0.12a	4.70 ± 0.38b
	MESA	0.08 ± 0.01a	0.06 ± 0.01a	0.08 ± 0.02a	0.08 ± 0.01a	0.08 ± 0.01a	0.06 ± 0.01a
JAs	JA	0.84 ± 0.05b	0.33 ± 0.06d	0.43 ± 0.03cd	0.93 ± 0.08b	1.67 ± 0.07a	0.55 ± 0.08c
	MEJA	2.45 ± 0.17b	1.27 ± 0.07d	2.09 ± 0.08c	2.93 ± 0.12a	0.95 ± 0.15d	0.42 ± 0.11e
GAs	GA1	ND	ND	ND	ND	ND	ND
	GA3	2.45 ± 0.11d	2.84 ± 0.07c	2.40 ± 0.05d	3.59 ± 0.05a	3.32 ± 0.04b	2.71 ± 0.07c
	GA4	0.08 ± 0.01bcd	0.12 ± 0.01a	0.05 ± 0.01d	0.11 ± 0.02ab	0.06 ± 0.01bcd	0.09 ± 0.01abc
	GA7	ND	ND	ND	ND	ND	ND
ACC	ACC	31.22 ± 0.51d	86.54 ± 2.23a	55.06 ± 0.86b	24.67 ± 0.10e	42.65 ± 2.55c	33.84 ± 3.55d
CTK	TZR	0.50 ± 0.01d	0.33 ± 0.00e	0.55 ± 0.00c	0.50 ± 0.00d	1.09 ± 0.01b	1.19 ± 0.01a

The three biological repeats in endogenous hormone were measured and the mean values ± SD (ng/g) are shown in each sample. Different letters in each line represent significantly differences (*p* < 0.05). ND: not detected.

## Supplementary Materials

The following are available online at https://www.mdpi.com/2073-4425/11/1/48/s1, Table S1: Primers used in this study; Table S2: The statistics of transcriptome data assembly quality and the statistics of unigene quality assembly; Table S3: List of GO terms related to stamen development genes; Table S4: GO enrichment with stamen development genes in SA pathway; Table S5: Selected reaction monitoring conditions for protonated or deprotonated plant hormones; Table S6: List of 459 DEGs related to hormones; Table S7: Up- and down-regulated DEGs of Profiles 0 and 7 in female and male flowers; Figure S1: The high conservation of similar functional genes in sequence (nucleic acid sequence or protein sequence) among different species, and functional annotation of unigene; Figure S2: The relationship of transcriptome samples and expression distribution from the stage of LC_M1/F1 to LC_M3/F3 were assessed by the Pearson correlation coefficient and boxplot; Figure S3: The enrichment analysis on KEGG pathway of DEGs in *L. cubeba*. Figure S4: The expression trend of 459 DEGs were analyzed by short time-series expression miner software (STEM) in female (LC_F1, LC_F2, LC_F3) and male flowers (LC_M1, LC_M2, LC_M3), respectively.
