# Supplementary material for: A Model of Hormonal Regulation of Stamen Abortion during Pre-Meiosis of Litsea cubeba
Source: Genes (Basel). 2019 Dec 31;11(1):48. doi: 10.3390/genes11010048 (PMC7017044; doi:10.3390/genes11010048)
Supplement: Supplementary file 1 [file genes-11-00048-s001.pdf]

Supplemental Figures and Tables

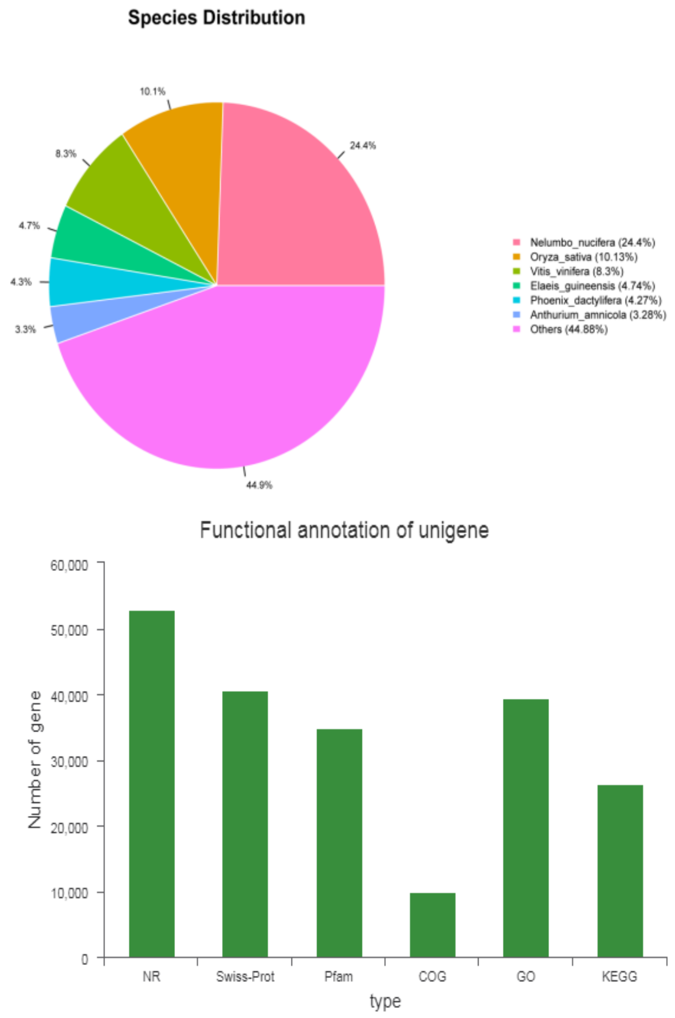

Figure S1. The high conservation of similar functional genes in sequence (nucleic acid sequence or protein sequence) among different species, and functional annotation of unigene.

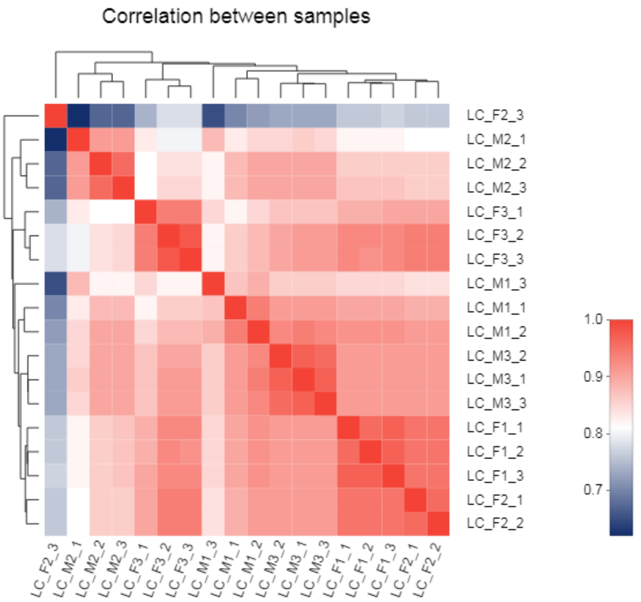

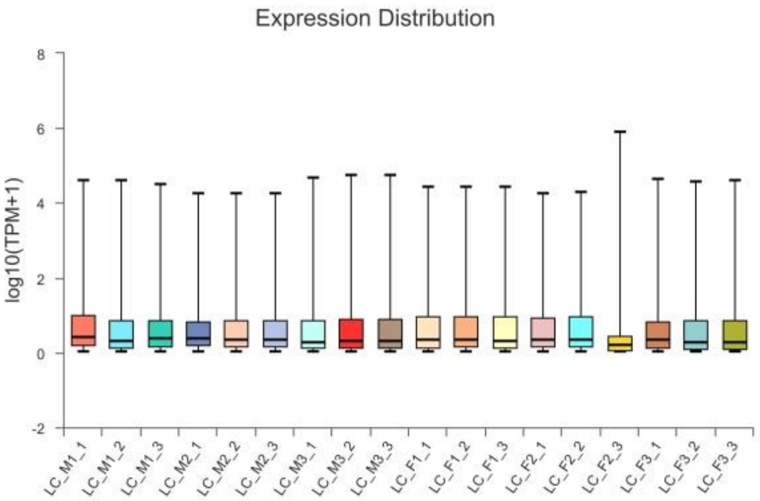

Figure S2. The relationship of transcriptome samples and expression distribution from the stage of LC_M1/F1 to LC_M3/F3 were assessed by the Pearson correlation coefficient and boxplot.

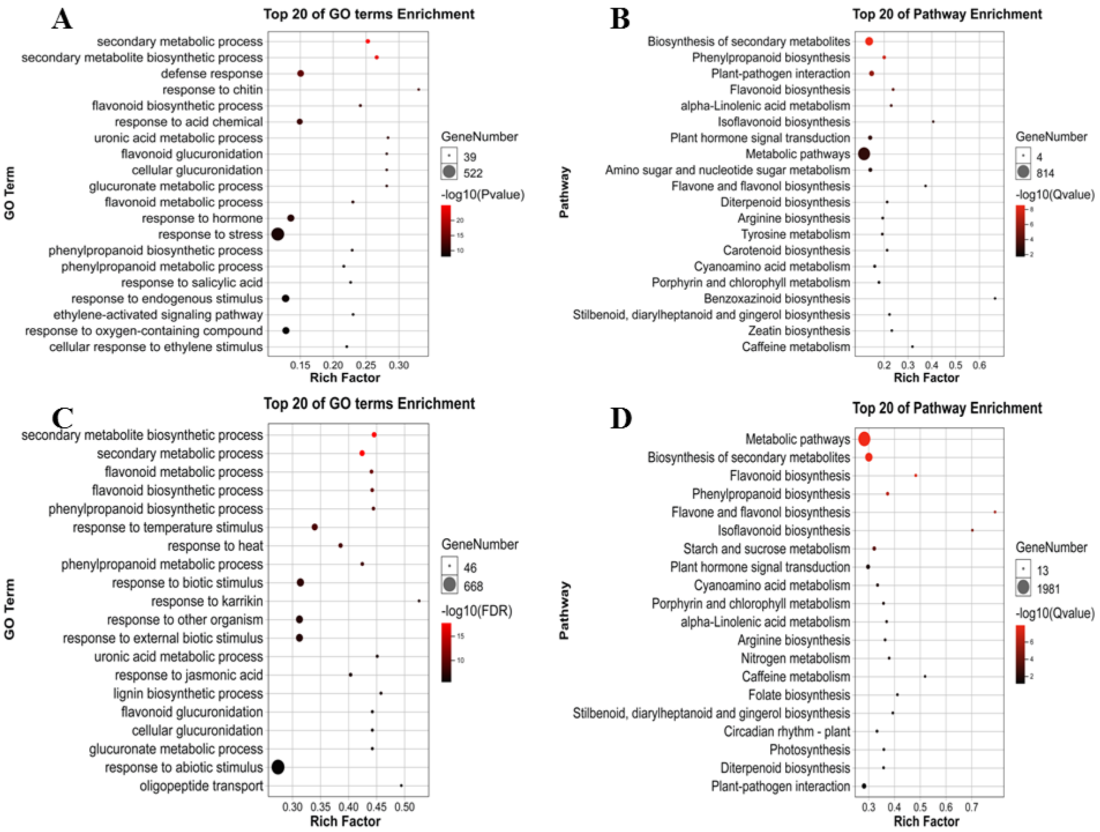

Figure S3. The enrichment analysis on KEGG pathway of DEGs in *L. cubeba.* (A) The top of 20 GO terms enrichment in LC_M1_vs_LC_F1; (B)The top of 20 pathway enrichment in LC_M1_vs_LC_F1; (C) The top of 20 GO terms enrichment in LC_M2_vs_LC_F2; (D)The top of 20 pathway enrichment in LC_M2_vs_LC_F2.

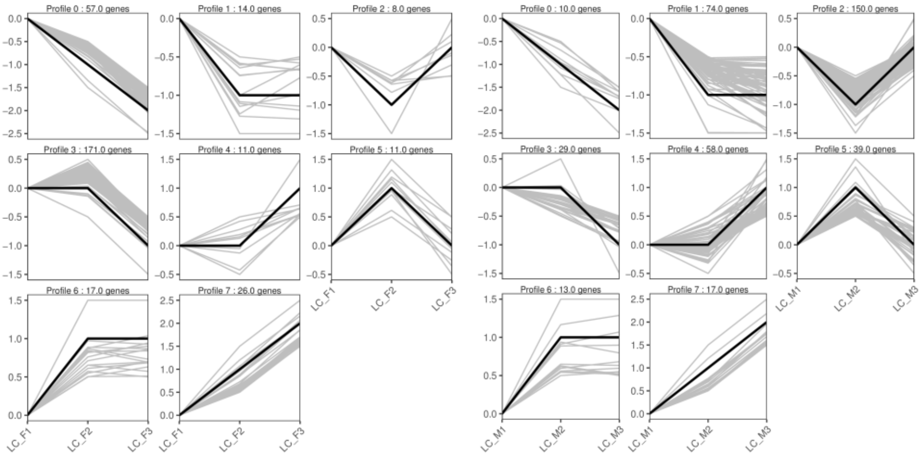

Figure S4. The expression trend of 459 DEGs were analyzed by Short Time-series Expression Miner software (STEM) in female (LC_F1, LC_F2, LC_F3) and male flowers (LC_M1, LC_M2,LC_M3) respectively.

Table S1. Primers used in this study.

Gene name	Forward primers (5'→3')	Reverse primers (5'→3')
ERF114	TCCGGACCTCCTTCAATATG	CGAAGCGAAGAAATCTCGAC
TGAL4	CAGCTGATCAACCAAAGCAA	TACAGGCACCAGTTGTGGAA
LG2	TGGCCGTGAAATCAGATGTC	CTGCCGGCGTCTTCCA
EMS1	GCGAATTGACCCAGAAAGAG	GGCAGCCATGTAGAGAGGAG
ARF6	TGCAGCAGCACTTAAGGAGA	ATGGAAGGTTGCTGGAATTG
ARF4	GTTGCAGGGGACACTTTTGT	TACATTATGCCGATGCCTGA
EXLB1	TTCTAGCCTTTGGCGTGGTT	TGCCTGGGTAGCTGCATGA
RAP2-7	TCTGGGATTGTGGAAAACAAGTG	CGCGCAGCAGCATGAG
ERF5	CCAGTCCTCACTCCTCCTCA	CTGTGGCCATCATCATCATC
ARF17	TTCCGAACTGGGTAACCTTG	AGCTCGACGAGGAACAGAAA
BAM1	CGGAGCTGAAGAATCTGACC	TGCTGCCAGTGAAGTTGTTC
STH21	ACGTGGGAAAGGAAGGACTGA	GCCAAGAGATGCCCTTCCA

Table S2-1. The statistics of transcriptome data assembly quality.

Sample	Raw_Reads	Raw_Bases	Valid_Reads	Valid_Bases	Valid%	Q20%	Q30%	GC%
LC_M1_1	39180882	5.88G	38011512	5.51G	97.02	97.27	92.96	46.42
LC_M1_2	60330556	9.05G	58993060	8.63G	97.78	98.04	94.52	46.74
LC_M1_3	54439902	8.17G	53088386	7.81G	97.52	98.42	95.74	47.08
LC_M2_1	49546420	7.43G	48523808	7.16G	97.94	98.71	96.40	46.65
LC_M2_2	57270748	8.59G	55881648	8.15G	97.57	97.82	94.03	46.60
LC_M2_3	54351942	8.15G	53023670	7.74G	97.56	97.90	94.23	46.55
LC_M3_1	51546652	7.73G	50305114	7.35G	97.59	97.99	94.40	46.84
LC_M3_2	46919256	7.04G	45730622	6.67G	97.47	97.83	94.04	46.75
LC_M3_3	50884620	7.63G	49587066	7.24G	97.45	97.90	94.18	46.89
LC_F1_1	44648754	6.70G	43499864	6.34G	97.43	97.72	93.86	46.77
LC_F1_2	43456966	6.52G	42269526	6.15G	97.27	97.67	93.73	46.73
LC_F1_3	46280162	6.94G	45127618	6.59G	97.51	97.88	94.15	46.81
LC_F2_1	48102374	7.22G	46878012	6.84G	97.45	97.90	94.20	46.50
LC_F2_2	43959482	6.59G	42675424	6.20G	97.08	97.58	93.55	46.68
LC_F2_3	54601944	8.21G	53058238	7.76G	97.17	97.93	94.19	51.05
LC_F3_1	40516372	6.08G	39669852	5.85G	97.91	98.64	96.27	47.07
LC_F3_2	55506094	8.33G	54142214	7.91G	97.54	97.94	94.26	47.02
LC_F3_3	50405390	7.56G	49227978	7.20G	97.66	98.00	94.42	47.17

Table S2-2. The statistics of unigenequality assembly.

Index	All	GC%	Min Length	Median Length	Max Length	Total Assembled Bases	N50
Transcript	209267	42.01	201	564	33543	200656300	1614
Gene	103921	42.30	201	428	33543	83413559	1412

Table S3. List of GO terms related to stamen development genes.

GO ID	GO Terms	Gene ID
GO:0048466	androecium development	TRINITY_DN32453_c0_g2;TRINITY_DN33546_c1_g1;TRINITY_DN35238_c0_g1;TRINITY_DN37902_c1_g1;TRINITY_DN43012_c0_g6;TRINITY_DN45981_c3_g1;TRINITY_DN46311_c3_g3;TRINITY_DN46311_c3_g4;TRINITY_DN47562_c1_g6;TRINITY_DN47562_c1_g1
GO:0048443	stamen development	TRINITY_DN32453_c0_g2;TRINITY_DN33546_c1_g1;TRINITY_DN35238_c0_g1;TRINITY_DN37902_c1_g1;TRINITY_DN43012_c0_g6;TRINITY_DN45981_c3_g1;TRINITY_DN46311_c3_g3;TRINITY_DN46311_c3_g4;TRINITY_DN47562_c1_g6;TRINITY_DN47562_c1_g1
GO:0048455	stamen formation	TRINITY_DN35238_c0_g1;TRINITY_DN43012_c0_g6
GO:0048448	stamen morphogenesis	TRINITY_DN35238_c0_g1;TRINITY_DN43012_c0_g6
GO:0048653	anther development	TRINITY_DN32453_c0_g2;TRINITY_DN33546_c1_g1;TRINITY_DN35238_c0_g1;TRINITY_DN37902_c1_g1;TRINITY_DN43012_c0_g6;TRINITY_DN45981_c3_g1;TRINITY_DN46311_c3_g3;TRINITY_DN46311_c3_g4;TRINITY_DN47562_c1_g6;TRINITY_DN47562_c1_g1
GO:0048656	anther wall tapetum formation	TRINITY_DN35238_c0_g1;TRINITY_DN43012_c0_g6
GO:0010234	anther wall tapetum cell fate specification	TRINITY_DN43012_c0_g6
GO:0048657	anther wall tapetum cell differentiation	TRINITY_DN35238_c0_g1;TRINITY_DN43012_c0_g6
GO:0048654	anther morphogenesis	TRINITY_DN35238_c0_g1;TRINITY_DN43012_c0_g6
GO:0048655	anther wall tapetum morphogenesis	TRINITY_DN35238_c0_g1;TRINITY_DN43012_c0_g6
GO:0048658	anther wall tapetum development	TRINITY_DN35238_c0_g1;TRINITY_DN43012_c0_g6
GO:0010152	pollen maturation	TRINITY_DN27555_c0_g1;TRINITY_DN48298_c0_g9
GO:0009555	pollen development	TRINITY_DN27555_c0_g1;TRINITY_DN33325_c0_g1;TRINITY_DN33546_c1_g1;TRINITY_DN35238_c0_g1;TRINITY_DN35249_c0_g4;TRINITY_DN43012_c0_g6;TRINITY_DN44001_c0_g3;TRINITY_DN48298_c0_g9
GO:0010584	pollen exine formation	TRINITY_DN35238_c0_g1
GO:0010208	pollen wall assembly	TRINITY_DN35238_c0_g1

Table S4. GO enrichment with stamen development genes in SA pathway.

GO Term	Gene ID	Name
GO:0009908	TRINITY_DN38311_c0_g2	HULK2
GO:0009908	TRINITY_DN40178_c0_g1	ARF8
GO:0009791	TRINITY_DN37137_c1_g3	FER
GO:0009555	TRINITY_DN40552_c3_g2	FBL17
GO:0010584	TRINITY_DN39537_c0_g1	A6
GO:0048653	TRINITY_DN37902_c1_g2	SPL18
GO:0009846	TRINITY_DN33045_c0_g1	APY1
GO:0009860	TRINITY_DN45582_c0_g1	MIRO1
GO:0080092	TRINITY_DN44636_c1_g1	ROPGEF14
GO:0048443	TRINITY_DN33496_c1_g7	GRP-2
GO:0048509	TRINITY_DN38362_c0_g3	AHK4
GO:0010500	TRINITY_DN32445_c0_g1	HEC3
GO:0010152	TRINITY_DN48298_c0_g10	SERK2
GO:0009555	TRINITY_DN47954_c1_g3	FAB1B
GO:0048544	TRINITY_DN42826_c1_g1	RAD6
GO:0009555	TRINITY_DN47954_c0_g1	FAB1B
GO:0009555	TRINITY_DN33888_c0_g10	FAB1B
GO:0016021	TRINITY_DN37667_c1_g3	GALT2
GO:0009908	TRINITY_DN44290_c0_g2	FES1
GO:0009556	TRINITY_DN42428_c3_g2	FH20
GO:0010183	TRINITY_DN41048_c0_g1	MIK2
GO:0009555	TRINITY_DN45368_c0_g1	CINV1
GO:0048868	TRINITY_DN46248_c1_g2	L1
GO:0009555	TRINITY_DN46568_c0_g3	NEDD1
GO:0009555	TRINITY_DN32842_c1_g1	FAB1B
GO:0009910	TRINITY_DN38304_c1_g1	ARP6
GO:0009908	TRINITY_DN42948_c2_g3	HAG1
GO:0009555	TRINITY_DN38204_c0_g5	Npc1
GO:0009908	TRINITY_DN45206_c2_g2	ARF5
GO:0010584	TRINITY_DN38174_c0_g1	C4
GO:0009846	TRINITY_DN45119_c0_g2	IP5P13
GO:0009556	TRINITY_DN42336_c1_g6	FH6
GO:0009556	TRINITY_DN33546_c1_g1	SPL8
GO:0048236	TRINITY_DN39501_c1_g2	MPS1
GO:0010584	TRINITY_DN44666_c1_g4	APY7
GO:0009555	TRINITY_DN38953_c1_g1	NFYA9
GO:0009911	TRINITY_DN35238_c0_g1	CURL3
GO:0009860	TRINITY_DN44018_c1_g1	SAB
GO:0010228	TRINITY_DN41250_c0_g2	ATXR3
GO:0009555	TRINITY_DN36467_c1_g1	XPO1
GO:0010483	TRINITY_DN16559_c0_g1	OST3B
GO:0090406	TRINITY_DN44876_c0_g6	LOC10
GO:0009846	TRINITY_DN37105_c1_g6	SEC8
GO:0009555	TRINITY_DN21282_c0_g1	LOC11

Table S5. Selected reaction monitoring conditions for protonated or deprotonated plant hormones ($[M+H]^+$ or$[M-H]^-$).

Name	Electrode	Precursor ions(m/z)	Product ions(m/z)	Clustering voltage (v)	Collision energy (v)
GA1	-	347.4	259.2/273.1	-25	-28/-26
GA3	-	345.2	143.0/239.2	-80	-30/-33
JA	-	209.2	58.9	-54	-16
MEJA	-	225.1	151.1/193.1/133	-50	-16/-10/-18
MESA	-	153.0	121.0/93.1/65.0	-131	-24/-39/-48
SA	-	137	92.9/65	-50	-20/-39
GA4	-	331.4	243.2/213.1	-131	-24/-39
GA7	-	329.2	223.2/241.1	-89	-38/-22
TZR	+	352.3	220.2/136/202.1	90	25/40/32
IAA	+	176.2	129.8/102.9	65	12/42
ACC	+	102.1	55.9/84.0	50	2/10

Table S6. List of 459 DEGs related to hormone.

Hormone	Gene ID	KO Description
**IAA**	TRINITY_DN43638_c0_g3	AUX1, LAX; auxin influx carrier (AUX1 LAX family)
	TRINITY_DN36415_c0_g2	AUX1, LAX; auxin influx carrier (AUX1 LAX family)
	TRINITY_DN45853_c0_g7	AUX1, LAX; auxin influx carrier (AUX1 LAX family)
	TRINITY_DN36415_c0_g5	AUX1, LAX; auxin influx carrier (AUX1 LAX family)
	TRINITY_DN44427_c1_g2	AUX1, LAX; auxin influx carrier (AUX1 LAX family)
	TRINITY_DN37538_c0_g1	AUX1, LAX; auxin influx carrier (AUX1 LAX family)
	TRINITY_DN42182_c0_g3	AUX1, LAX; auxin influx carrier (AUX1 LAX family)
	TRINITY_DN34895_c0_g1	IAA; auxin-responsive protein IAA
	TRINITY_DN33821_c0_g6	IAA; auxin-responsive protein IAA
	TRINITY_DN39053_c0_g9	IAA; auxin-responsive protein IAA
	TRINITY_DN33821_c0_g2	IAA; auxin-responsive protein IAA
	TRINITY_DN31729_c1_g1	IAA; auxin-responsive protein IAA
	TRINITY_DN33167_c0_g1	IAA; auxin-responsive protein IAA
	TRINITY_DN48595_c3_g1	IAA; auxin-responsive protein IAA
	TRINITY_DN30139_c0_g1	IAA; auxin-responsive protein IAA
	TRINITY_DN48172_c1_g1	IAA; auxin-responsive protein IAA
	TRINITY_DN33914_c0_g1	IAA; auxin-responsive protein IAA
	TRINITY_DN45579_c0_g3	IAA; auxin-responsive protein IAA
	TRINITY_DN33123_c3_g1	IAA; auxin-responsive protein IAA
	TRINITY_DN44119_c0_g1	IAA; auxin-responsive protein IAA
	TRINITY_DN45399_c0_g1	TIR1; transport inhibitor response 1
	TRINITY_DN33268_c0_g1	TIR1; transport inhibitor response 1
	TRINITY_DN46765_c0_g3	K14486, ARF; auxin response factor
	TRINITY_DN46765_c0_g7	K14486, ARF; auxin response factor
	TRINITY_DN41025_c2_g2	K14486, ARF; auxin response factor
	TRINITY_DN48307_c2_g1	K14486, ARF; auxin response factor
	TRINITY_DN34502_c1_g3	K14486, ARF; auxin response factor
	TRINITY_DN45206_c2_g2	K14486, ARF; auxin response factor
	TRINITY_DN35441_c0_g4	K14486, ARF; auxin response factor
	TRINITY_DN39047_c0_g3	K14486, ARF; auxin response factor
	TRINITY_DN42693_c0_g4	K14486, ARF; auxin response factor
	TRINITY_DN44779_c2_g2	K14486, ARF; auxin response factor
	TRINITY_DN36058_c2_g11	K14486, ARF; auxin response factor
	TRINITY_DN46815_c1_g3	K14486, ARF; auxin response factor
	TRINITY_DN41193_c1_g1	K14486, ARF; auxin response factor
	TRINITY_DN35494_c0_g3	K14486, ARF; auxin response factor
	TRINITY_DN33930_c2_g1	K14486, ARF; auxin response factor
	TRINITY_DN40178_c0_g2	K14486, ARF; auxin response factor
	TRINITY_DN41193_c1_g5	K14486, ARF; auxin response factor
	TRINITY_DN38814_c1_g4	K14486, ARF; auxin response factor
	TRINITY_DN38814_c1_g2	K14486, ARF; auxin response factor
	TRINITY_DN35441_c0_g7	K14486, ARF; auxin response factor
	TRINITY_DN48164_c1_g4	K14486, ARF; auxin response factor
	TRINITY_DN36058_c2_g1	K14486, ARF; auxin response factor
	TRINITY_DN36330_c0_g1	K14486, ARF; auxin response factor
	TRINITY_DN47842_c1_g2	K14486, ARF; auxin response factor
	TRINITY_DN45771_c0_g3	K14486, ARF; auxin response factor
	TRINITY_DN48307_c2_g2	K14486, ARF; auxin response factor
	TRINITY_DN40178_c0_g1	K14486, ARF; auxin response factor

	TRINITY_DN44080_c1_g2	GH3; auxin responsive GH3 gene family
	TRINITY_DN46627_c1_g1	GH3; auxin responsive GH3 gene family
	TRINITY_DN42192_c0_g1	GH3; auxin responsive GH3 gene family
	TRINITY_DN32879_c0_g5	GH3; auxin responsive GH3 gene family
	TRINITY_DN44080_c1_g4	GH3; auxin responsive GH3 gene family
	TRINITY_DN41774_c0_g1	GH3; auxin responsive GH3 gene family
	TRINITY_DN36428_c0_g3	GH3; auxin responsive GH3 gene family
	TRINITY_DN44451_c1_g3	GH3; auxin responsive GH3 gene family
	TRINITY_DN39675_c1_g1	GH3; auxin responsive GH3 gene family
	TRINITY_DN41724_c0_g1	GH3; auxin responsive GH3 gene family
	TRINITY_DN42184_c5_g2	SAUR; SAUR family protein
	TRINITY_DN43828_c1_g3	SAUR; SAUR family protein
	TRINITY_DN44985_c2_g1	SAUR; SAUR family protein
	TRINITY_DN29586_c0_g1	SAUR; SAUR family protein
	TRINITY_DN43785_c0_g1	SAUR; SAUR family protein
	TRINITY_DN40505_c0_g1	SAUR; SAUR family protein
	TRINITY_DN36575_c2_g1	SAUR; SAUR family protein
	TRINITY_DN31085_c0_g1	SAUR; SAUR family protein
	TRINITY_DN31433_c0_g1	SAUR; SAUR family protein
	TRINITY_DN37207_c0_g8	SAUR; SAUR family protein
	TRINITY_DN45035_c0_g5	SAUR; SAUR family protein
	TRINITY_DN45035_c0_g3	SAUR; SAUR family protein
	TRINITY_DN44242_c2_g1	SAUR; SAUR family protein
	TRINITY_DN33819_c1_g3	SAUR; SAUR family protein
	TRINITY_DN41416_c2_g3	SAUR; SAUR family protein
	TRINITY_DN33819_c1_g7	SAUR; SAUR family protein
	TRINITY_DN48237_c6_g1	SAUR; SAUR family protein
	TRINITY_DN45753_c5_g1	SAUR; SAUR family protein
	TRINITY_DN48564_c1_g5	SAUR; SAUR family protein
	TRINITY_DN31869_c0_g1	SAUR; SAUR family protein
	TRINITY_DN42184_c5_g1	SAUR; SAUR family protein
	TRINITY_DN48564_c1_g1	SAUR; SAUR family protein
	TRINITY_DN29124_c0_g1	SAUR; SAUR family protein
	TRINITY_DN37593_c1_g1	SAUR; SAUR family protein
	TRINITY_DN38212_c1_g2	SAUR; SAUR family protein
	TRINITY_DN41416_c2_g5	SAUR; SAUR family protein
	TRINITY_DN23467_c0_g1	SAUR; SAUR family protein
	TRINITY_DN36486_c1_g2	SAUR; SAUR family protein
	TRINITY_DN41416_c2_g1	SAUR; SAUR family protein
	TRINITY_DN41416_c2_g4	SAUR; SAUR family protein
**CTK**	TRINITY_DN42091_c0_g6	AHK2_3_4; arabidopsishistidine kinase 2/3/4 (cytokinin receptor)
	TRINITY_DN38362_c0_g3	AHK2_3_4; arabidopsishistidine kinase 2/3/4 (cytokinin receptor)
	TRINITY_DN45954_c0_g4	AHK2_3_4; arabidopsishistidine kinase 2/3/4 (cytokinin receptor)
	TRINITY_DN43522_c2_g5	AHK2_3_4; arabidopsishistidine kinase 2/3/4 (cytokinin receptor)
	TRINITY_DN45954_c0_g1	AHK2_3_4; arabidopsishistidine kinase 2/3/4 (cytokinin receptor)
	TRINITY_DN40127_c1_g1	AHP; histidine-containing phosphotransferpeotein

	TRINITY_DN43499_c4_g1	AHP; histidine-containing phosphotransferpeotein
	TRINITY_DN31066_c0_g1	AHP; histidine-containing phosphotransferpeotein
	TRINITY_DN34396_c0_g7	AHP; histidine-containing phosphotransferpeotein
	TRINITY_DN44950_c2_g1	ARR-B; two-component response regulator ARR-B family
	TRINITY_DN37937_c0_g5	ARR-B; two-component response regulator ARR-B family
	TRINITY_DN45736_c0_g4	ARR-B; two-component response regulator ARR-B family
	TRINITY_DN30266_c0_g1	ARR-B; two-component response regulator ARR-B family
	TRINITY_DN32538_c0_g1	ARR-B; two-component response regulator ARR-B family
	TRINITY_DN38678_c1_g1	ARR-B; two-component response regulator ARR-B family
	TRINITY_DN37799_c1_g5	ARR-B; two-component response regulator ARR-B family
	TRINITY_DN43808_c0_g5	ARR-B; two-component response regulator ARR-B family
	TRINITY_DN36642_c0_g9	ARR-B; two-component response regulator ARR-B family
	TRINITY_DN38678_c1_g5	ARR-B; two-component response regulator ARR-B family
	TRINITY_DN33779_c2_g1	ARR-B; two-component response regulator ARR-B family
	TRINITY_DN33126_c4_g2	ARR-A; two-component response regulator ARR-A family
	TRINITY_DN33126_c4_g6	ARR-A; two-component response regulator ARR-A family
	TRINITY_DN47708_c1_g5	ARR-A; two-component response regulator ARR-A family
	TRINITY_DN34078_c0_g2	ARR-A; two-component response regulator ARR-A family
	TRINITY_DN38410_c0_g2	ARR-A; two-component response regulator ARR-A family
	TRINITY_DN32344_c0_g1	ARR-A; two-component response regulator ARR-A family
**GA**	TRINITY_DN34643_c2_g2	GID1; gibberellin receptor GID1
	TRINITY_DN46161_c1_g2	GID1; gibberellin receptor GID1
	TRINITY_DN35027_c0_g2	GID1; gibberellin receptor GID1
	TRINITY_DN38964_c0_g1	GID1; gibberellin receptor GID1
	TRINITY_DN46161_c1_g1	GID1; gibberellin receptor GID1
	TRINITY_DN38205_c0_g2	GID1; gibberellin receptor GID1
	TRINITY_DN35025_c1_g2	GID1; gibberellin receptor GID1
	TRINITY_DN36539_c0_g1	GID1; gibberellin receptor GID1
	TRINITY_DN38964_c0_g2	GID1; gibberellin receptor GID1
	TRINITY_DN31125_c0_g1	DELLA; DELLA protein
	TRINITY_DN38919_c0_g1	DELLA; DELLA protein
	TRINITY_DN35822_c0_g4	DELLA; DELLA protein
	TRINITY_DN34861_c0_g2	DELLA; DELLA protein
	TRINITY_DN26483_c0_g1	DELLA; DELLA protein

	TRINITY_DN31079_c0_g1	DELLA; DELLA protein
	TRINITY_DN46599_c0_g1	DELLA; DELLA protein
	TRINITY_DN32817_c0_g1	DELLA; DELLA protein
	TRINITY_DN38429_c3_g7	DELLA; DELLA protein
	TRINITY_DN35046_c0_g5	DELLA; DELLA protein
	TRINITY_DN30717_c0_g2	DELLA; DELLA protein
	TRINITY_DN38732_c5_g1	DELLA; DELLA protein
	TRINITY_DN35936_c1_g5	DELLA; DELLA protein
	TRINITY_DN27178_c0_g1	GID2, SLY1; F-box protein GID2
	TRINITY_DN32468_c0_g1	GID2, SLY1; F-box protein GID2
	TRINITY_DN32015_c0_g1	GID2, SLY1; F-box protein GID2
	TRINITY_DN41833_c2_g1	GID2, SLY1; F-box protein GID2
	TRINITY_DN42846_c1_g1	PIF4; phytochrome-interacting factor 4
	TRINITY_DN47831_c0_g1	PIF4; phytochrome-interacting factor 4
	TRINITY_DN35730_c3_g10	PIF4; phytochrome-interacting factor 4
	TRINITY_DN44950_c2_g2	PIF4; phytochrome-interacting factor 4
	TRINITY_DN35730_c3_g5	PIF4; phytochrome-interacting factor 4
	TRINITY_DN36292_c0_g5	PIF4; phytochrome-interacting factor 4
	TRINITY_DN39368_c0_g2	PIF4; phytochrome-interacting factor 4
	TRINITY_DN31876_c0_g1	PIF4; phytochrome-interacting factor 4
	TRINITY_DN46794_c1_g5	PIF4; phytochrome-interacting factor 4
	TRINITY_DN42374_c2_g1	PIF4; phytochrome-interacting factor 4
	TRINITY_DN45157_c0_g2	PIF3; phytochrome-interacting factor 3
	TRINITY_DN43198_c0_g3	PIF3; phytochrome-interacting factor 3
	TRINITY_DN45157_c0_g6	PIF3; phytochrome-interacting factor 3
	TRINITY_DN35485_c0_g1	PIF3; phytochrome-interacting factor 3
	TRINITY_DN32735_c0_g1	PIF3; phytochrome-interacting factor 3
	TRINITY_DN46794_c0_g1	PIF3; phytochrome-interacting factor 3
	TRINITY_DN40112_c0_g1	PIF3; phytochrome-interacting factor 3
	TRINITY_DN32445_c0_g1	PIF3; phytochrome-interacting factor 3
	TRINITY_DN43774_c2_g1	PIF3; phytochrome-interacting factor 3
	TRINITY_DN36256_c1_g1	PIF3; phytochrome-interacting factor 3
	TRINITY_DN39197_c2_g2	PIF3; phytochrome-interacting factor 3
	TRINITY_DN35729_c0_g1	PIF3; phytochrome-interacting factor 3
	TRINITY_DN28010_c0_g2	PIF3; phytochrome-interacting factor 3
	TRINITY_DN47444_c0_g1	PIF3; phytochrome-interacting factor 3
	TRINITY_DN29286_c0_g1	PIF3; phytochrome-interacting factor 3
	TRINITY_DN39418_c1_g4	PIF3; phytochrome-interacting factor 3
**ABA**	TRINITY_DN43208_c0_g4	ABF; ABA responsive element binding factor
	TRINITY_DN40597_c0_g1	ABF; ABA responsive element binding factor
	TRINITY_DN45167_c0_g1	ABF; ABA responsive element binding factor
	TRINITY_DN45132_c2_g1	ABF; ABA responsive element binding factor
	TRINITY_DN30413_c0_g1	ABF; ABA responsive element binding factor
	TRINITY_DN45101_c1_g4	ABF; ABA responsive element binding factor
	TRINITY_DN45167_c0_g2	ABF; ABA responsive element binding factor
	TRINITY_DN42404_c0_g1	ABF; ABA responsive element binding factor
	TRINITY_DN31778_c0_g1	ABF; ABA responsive element binding factor
	TRINITY_DN27555_c0_g1	ABF; ABA responsive element binding factor
	TRINITY_DN34170_c2_g4	PYL; abscisic acid receptor PYR/PYL family
	TRINITY_DN41397_c0_g5	PYL; abscisic acid receptor PYR/PYL family
	TRINITY_DN46185_c0_g3	PYL; abscisic acid receptor PYR/PYL family

	TRINITY_DN16026_c0_g1	PYL; abscisic acid receptor PYR/PYL family
	TRINITY_DN48042_c4_g3	PYL; abscisic acid receptor PYR/PYL family
	TRINITY_DN31755_c0_g1	PYL; abscisic acid receptor PYR/PYL family
	TRINITY_DN48042_c4_g2	PYL; abscisic acid receptor PYR/PYL family
	TRINITY_DN31174_c0_g1	PYL; abscisic acid receptor PYR/PYL family
	TRINITY_DN40399_c0_g2	SNRK2; serine/threonine-protein kinase SRK2
	TRINITY_DN37658_c0_g2	SNRK2; serine/threonine-protein kinase SRK2
	TRINITY_DN34069_c0_g1	SNRK2; serine/threonine-protein kinase SRK2
	TRINITY_DN35928_c0_g5	SNRK2; serine/threonine-protein kinase SRK2
	TRINITY_DN39876_c1_g1	SNRK2; serine/threonine-protein kinase SRK2
	TRINITY_DN35928_c0_g3	SNRK2; serine/threonine-protein kinase SRK2
	TRINITY_DN45898_c0_g1	SNRK2; serine/threonine-protein kinase SRK2
	TRINITY_DN37333_c4_g1	SNRK2; serine/threonine-protein kinase SRK2
	TRINITY_DN41342_c0_g5	SNRK2; serine/threonine-protein kinase SRK2
	TRINITY_DN35928_c1_g1	SNRK2; serine/threonine-protein kinase SRK2
	TRINITY_DN34069_c0_g2	SNRK2; serine/threonine-protein kinase SRK2
	TRINITY_DN44280_c0_g2	SNRK2; serine/threonine-protein kinase SRK2
	TRINITY_DN44280_c0_g1	SNRK2; serine/threonine-protein kinase SRK2
	TRINITY_DN37281_c0_g1	SNRK2; serine/threonine-protein kinase SRK2
	TRINITY_DN43444_c1_g1	SNRK2; serine/threonine-protein kinase SRK2
	TRINITY_DN42746_c3_g3	SNRK2; serine/threonine-protein kinase SRK2
	TRINITY_DN44956_c0_g4	SNRK2; serine/threonine-protein kinase SRK2
	TRINITY_DN38567_c1_g2	SNRK2; serine/threonine-protein kinase SRK2
	TRINITY_DN39839_c0_g1	SNRK2; serine/threonine-protein kinase SRK2
	TRINITY_DN35928_c0_g1	SNRK2; serine/threonine-protein kinase SRK2
	TRINITY_DN37658_c0_g3	SNRK2; serine/threonine-protein kinase SRK2
	TRINITY_DN42074_c0_g1	SNRK2; serine/threonine-protein kinase SRK2
	TRINITY_DN31584_c0_g1	SNRK2; serine/threonine-protein kinase SRK2
	TRINITY_DN33113_c0_g2	SNRK2; serine/threonine-protein kinase SRK2
	TRINITY_DN37925_c1_g1	SNRK2; serine/threonine-protein kinase SRK2
	TRINITY_DN39876_c1_g2	SNRK2; serine/threonine-protein kinase SRK2
	TRINITY_DN42746_c3_g7	SNRK2; serine/threonine-protein kinase SRK2
**ETH**	TRINITY_DN33232_c0_g1	ETR, ERS; ethylene receptor
	TRINITY_DN48079_c4_g1	ETR, ERS; ethylene receptor
	TRINITY_DN46342_c0_g3	CTR1; serine/threonine-protein kinase CTR1
	TRINITY_DN44870_c0_g1	CTR1; serine/threonine-protein kinase CTR1
	TRINITY_DN46105_c0_g3	CTR1; serine/threonine-protein kinase CTR1
	TRINITY_DN36149_c0_g3	CTR1; serine/threonine-protein kinase CTR1
	TRINITY_DN44945_c2_g1	CTR1; serine/threonine-protein kinase CTR1
	TRINITY_DN38693_c0_g1	CTR1; serine/threonine-protein kinase CTR1
	TRINITY_DN47241_c2_g2	CTR1; serine/threonine-protein kinase CTR1
	TRINITY_DN37654_c2_g5	CTR1; serine/threonine-protein kinase CTR1
	TRINITY_DN45443_c0_g1	CTR1; serine/threonine-protein kinase CTR1
	TRINITY_DN34839_c1_g5	CTR1; serine/threonine-protein kinase CTR1
	TRINITY_DN40260_c2_g2	CTR1; serine/threonine-protein kinase CTR1
	TRINITY_DN35552_c0_g4	CTR1; serine/threonine-protein kinase CTR1
	TRINITY_DN45443_c0_g2	CTR1; serine/threonine-protein kinase CTR1
	TRINITY_DN40852_c0_g2	CTR1; serine/threonine-protein kinase CTR1
	TRINITY_DN40171_c0_g2	CTR1; serine/threonine-protein kinase CTR1
	TRINITY_DN39689_c1_g2	CTR1; serine/threonine-protein kinase CTR1
	TRINITY_DN35061_c0_g1	CTR1; serine/threonine-protein kinase CTR1

	TRINITY_DN44503_c3_g2	CTR1; serine/threonine-protein kinase CTR1
	TRINITY_DN35249_c0_g4	MPK6; mitogen-activated protein kinase 6
	TRINITY_DN47490_c1_g2	MPK6; mitogen-activated protein kinase 6
	TRINITY_DN41074_c2_g4	MPK6; mitogen-activated protein kinase 6
	TRINITY_DN47738_c0_g1	MPK6; mitogen-activated protein kinase 6
	TRINITY_DN33325_c0_g1	MPK6; mitogen-activated protein kinase 6
	TRINITY_DN44001_c0_g3	MPK6; mitogen-activated protein kinase 6
	TRINITY_DN39324_c0_g1	MPK6; mitogen-activated protein kinase 6
	TRINITY_DN43413_c0_g3	MPK6; mitogen-activated protein kinase 6
	TRINITY_DN45966_c0_g4	MPK6; mitogen-activated protein kinase 6
	TRINITY_DN42852_c1_g2	EIN2; ethylene-insensitive protein 2
	TRINITY_DN33711_c2_g16	EIN2; ethylene-insensitive protein 2
	TRINITY_DN37135_c1_g4	EIN2; ethylene-insensitive protein 2
	TRINITY_DN42678_c3_g6	EIN2; ethylene-insensitive protein 2
	TRINITY_DN41979_c1_g1	EIN3; ethylene-insensitive protein 3
	TRINITY_DN37649_c0_g6	EIN3; ethylene-insensitive protein 3
	TRINITY_DN41230_c0_g3	EIN3; ethylene-insensitive protein 3
	TRINITY_DN41230_c0_g2	EIN3; ethylene-insensitive protein 3
	TRINITY_DN44414_c0_g2	EIN3; ethylene-insensitive protein 3
	TRINITY_DN43293_c1_g1	ERF1; ethylene-responsive transcription factor 1
	TRINITY_DN44843_c1_g4	ERF1; ethylene-responsive transcription factor 1
	TRINITY_DN34785_c0_g3	ERF1; ethylene-responsive transcription factor 1
	TRINITY_DN36854_c2_g3	ERF1; ethylene-responsive transcription factor 1
	TRINITY_DN44023_c0_g2	ERF1; ethylene-responsive transcription factor 1
	TRINITY_DN47977_c6_g1	ERF1; ethylene-responsive transcription factor 1
	TRINITY_DN45336_c4_g2	ERF1; ethylene-responsive transcription factor 1
	TRINITY_DN36402_c1_g3	ERF2; ethylene-responsive transcription factor 2
	TRINITY_DN39857_c0_g1	ERF2; ethylene-responsive transcription factor 2
	TRINITY_DN43821_c0_g5	ERF2; ethylene-responsive transcription factor 2
	TRINITY_DN34785_c0_g2	ERF2; ethylene-responsive transcription factor 2
	TRINITY_DN36402_c2_g1	ERF2; ethylene-responsive transcription factor 2
	TRINITY_DN33574_c1_g5	ERF2; ethylene-responsive transcription factor 2
	TRINITY_DN36853_c0_g6	ERF2; ethylene-responsive transcription factor 2
	TRINITY_DN38148_c0_g1	ERF2; ethylene-responsive transcription factor 2
	TRINITY_DN33574_c0_g1	ERF2; ethylene-responsive transcription factor 2
	TRINITY_DN39180_c1_g1	MKK4_5; mitogen-activated protein kinase kinase 4/5
	TRINITY_DN39086_c2_g1	MKK4_5; mitogen-activated protein kinase kinase 4/5
**BR**	TRINITY_DN35765_c0_g1	BRI1; protein brassinosteroid insensitive 1
	TRINITY_DN46917_c3_g2	BRI1; protein brassinosteroid insensitive 1
	TRINITY_DN35850_c0_g2	BRI1; protein brassinosteroid insensitive 1
	TRINITY_DN34063_c3_g2	BRI1; protein brassinosteroid insensitive 1
	TRINITY_DN44178_c1_g6	BRI1; protein brassinosteroid insensitive 1
	TRINITY_DN45285_c0_g5	BRI1; protein brassinosteroid insensitive 1
	TRINITY_DN42476_c0_g1	BRI1; protein brassinosteroid insensitive 1
	TRINITY_DN37256_c0_g3	BRI1; protein brassinosteroid insensitive 1
	TRINITY_DN36648_c2_g4	BRI1; protein brassinosteroid insensitive 1
	TRINITY_DN41687_c0_g5	BRI1; protein brassinosteroid insensitive 1
	TRINITY_DN48347_c0_g2	BRI1; protein brassinosteroid insensitive 1
	TRINITY_DN43012_c0_g6	BRI1; protein brassinosteroid insensitive 1
	TRINITY_DN41514_c2_g1	BRI1; protein brassinosteroid insensitive 1
	TRINITY_DN36757_c0_g3	BRI1; protein brassinosteroid insensitive 1

TRINITY_DN35454_c0_g2	BRI1; protein brassinosteroid insensitive 1
TRINITY_DN39957_c0_g4	BRI1; protein brassinosteroid insensitive 1
TRINITY_DN44294_c0_g2	BRI1; protein brassinosteroid insensitive 1
TRINITY_DN43443_c2_g1	BRI1; protein brassinosteroid insensitive 1
TRINITY_DN45444_c1_g2	BRI1; protein brassinosteroid insensitive 1
TRINITY_DN37670_c3_g9	BRI1; protein brassinosteroid insensitive 1
TRINITY_DN40594_c0_g2	BRI1; protein brassinosteroid insensitive 1
TRINITY_DN48432_c2_g1	BRI1; protein brassinosteroid insensitive 1
TRINITY_DN45896_c0_g1	BRI1; protein brassinosteroid insensitive 1
TRINITY_DN46939_c3_g1	BRI1; protein brassinosteroid insensitive 1
TRINITY_DN35238_c0_g1	BRI1; protein brassinosteroid insensitive 1
TRINITY_DN45538_c1_g4	BRI1; protein brassinosteroid insensitive 1
TRINITY_DN43559_c2_g4	BRI1; protein brassinosteroid insensitive 1
TRINITY_DN36261_c0_g1	BRI1; protein brassinosteroid insensitive 1
TRINITY_DN48301_c3_g1	BRI1; protein brassinosteroid insensitive 1
TRINITY_DN39009_c0_g1	BRI1; protein brassinosteroid insensitive 1
TRINITY_DN47046_c0_g1	BRI1; protein brassinosteroid insensitive 1
TRINITY_DN44512_c0_g2	BRI1; protein brassinosteroid insensitive 1
TRINITY_DN35939_c4_g2	BRI1; protein brassinosteroid insensitive 1
TRINITY_DN33546_c1_g1	BRI1; protein brassinosteroid insensitive 1
TRINITY_DN37377_c1_g3	BRI1; protein brassinosteroid insensitive 1
TRINITY_DN46189_c0_g10	BRI1; protein brassinosteroid insensitive 1
TRINITY_DN43083_c4_g1	BRI1; protein brassinosteroid insensitive 1
TRINITY_DN37394_c1_g8	BRI1; protein brassinosteroid insensitive 1
TRINITY_DN39647_c1_g1	BRI1; protein brassinosteroid insensitive 1
TRINITY_DN38457_c1_g6	BRI1; protein brassinosteroid insensitive 1
TRINITY_DN39565_c0_g5	BRI1; protein brassinosteroid insensitive 1
TRINITY_DN36002_c0_g4	BRI1; protein brassinosteroid insensitive 1
TRINITY_DN46189_c0_g4	BRI1; protein brassinosteroid insensitive 1
TRINITY_DN44178_c1_g4	BRI1; protein brassinosteroid insensitive 1
TRINITY_DN34236_c1_g1	BRI1; protein brassinosteroid insensitive 1
TRINITY_DN38973_c0_g1	BRI1; protein brassinosteroid insensitive 1
TRINITY_DN47727_c0_g5	BRI1; protein brassinosteroid insensitive 1
TRINITY_DN46936_c1_g2	BRI1; protein brassinosteroid insensitive 1
TRINITY_DN39565_c0_g4	BRI1; protein brassinosteroid insensitive 1
TRINITY_DN35850_c0_g3	BRI1; protein brassinosteroid insensitive 1
TRINITY_DN35138_c0_g1	BRI1; protein brassinosteroid insensitive 1
TRINITY_DN45981_c3_g1	BRI1; protein brassinosteroid insensitive 1
TRINITY_DN39039_c6_g1	BRI1; protein brassinosteroid insensitive 1
TRINITY_DN35624_c0_g1	BRI1; protein brassinosteroid insensitive 1
TRINITY_DN47387_c1_g2	BRI1; protein brassinosteroid insensitive 1
TRINITY_DN45538_c1_g2	BRI1; protein brassinosteroid insensitive 1
TRINITY_DN32453_c0_g2	BRI1; protein brassinosteroid insensitive 1
TRINITY_DN41048_c0_g1	BRI1; protein brassinosteroid insensitive 1
TRINITY_DN37420_c1_g1	BRI1; protein brassinosteroid insensitive 1
TRINITY_DN34911_c1_g4	BRI1; protein brassinosteroid insensitive 1
TRINITY_DN47243_c0_g1	BRI1; protein brassinosteroid insensitive 1
TRINITY_DN46311_c3_g4	BRI1; protein brassinosteroid insensitive 1
TRINITY_DN39121_c0_g2	BRI1; protein brassinosteroid insensitive 1
TRINITY_DN45470_c2_g1	BRI1; protein brassinosteroid insensitive 1
TRINITY_DN47030_c2_g1	BRI1; protein brassinosteroid insensitive 1

	TRINITY_DN39404_c2_g3	BRI1; protein brassinosteroid insensitive 1
	TRINITY_DN34236_c1_g7	BRI1; protein brassinosteroid insensitive 1
	TRINITY_DN44335_c1_g1	BRI1; protein brassinosteroid insensitive 1
	TRINITY_DN37902_c1_g1	BRI1; protein brassinosteroid insensitive 1
	TRINITY_DN39708_c1_g1	BRI1; protein brassinosteroid insensitive 1
	TRINITY_DN32075_c0_g1	BRI1; protein brassinosteroid insensitive 1
	TRINITY_DN39906_c1_g8	BRI1; protein brassinosteroid insensitive 1
	TRINITY_DN46996_c2_g1	BRI1; protein brassinosteroid insensitive 1
	TRINITY_DN47387_c1_g1	BRI1; protein brassinosteroid insensitive 1
	TRINITY_DN46311_c3_g3	BRI1; protein brassinosteroid insensitive 1
	TRINITY_DN47243_c1_g1	BRI1; protein brassinosteroid insensitive 1
	TRINITY_DN47197_c1_g2	BRI1; protein brassinosteroid insensitive 1
	TRINITY_DN39565_c0_g2	BAK1; brassinosteroid insensitive 1-associated receptor kinase 1
	TRINITY_DN37853_c6_g1	BAK1; brassinosteroid insensitive 1-associated receptor kinase 1
	TRINITY_DN46458_c2_g1	BAK1; brassinosteroid insensitive 1-associated receptor kinase 1
	TRINITY_DN37116_c0_g2	BAK1; brassinosteroid insensitive 1-associated receptor kinase 1
	TRINITY_DN39105_c1_g2	BAK1; brassinosteroid insensitive 1-associated receptor kinase 1
	TRINITY_DN41405_c0_g5	BAK1; brassinosteroid insensitive 1-associated receptor kinase 1
	TRINITY_DN42818_c0_g1	BAK1; brassinosteroid insensitive 1-associated receptor kinase 1
	TRINITY_DN42938_c0_g1	BAK1; brassinosteroid insensitive 1-associated receptor kinase 1
	TRINITY_DN35188_c0_g2	BAK1; brassinosteroid insensitive 1-associated receptor kinase 1
	TRINITY_DN41883_c0_g1	BAK1; brassinosteroid insensitive 1-associated receptor kinase 1
	TRINITY_DN41883_c0_g2	BAK1; brassinosteroid insensitive 1-associated receptor kinase 1
	TRINITY_DN44591_c0_g1	BAK1; brassinosteroid insensitive 1-associated receptor kinase 1
	TRINITY_DN38877_c0_g1	BAK1; brassinosteroid insensitive 1-associated receptor kinase 1
	TRINITY_DN44105_c1_g1	BAK1; brassinosteroid insensitive 1-associated receptor kinase 1
	TRINITY_DN48298_c0_g9	BAK1; brassinosteroid insensitive 1-associated receptor kinase 1
	TRINITY_DN44735_c2_g1	BAK1; brassinosteroid insensitive 1-associated receptor kinase 1
	TRINITY_DN46222_c0_g3	BAK1; brassinosteroid insensitive 1-associated receptor kinase 1
	TRINITY_DN47721_c0_g1	BAK1; brassinosteroid insensitive 1-associated receptor kinase 1
	TRINITY_DN42811_c0_g2	BAK1; brassinosteroid insensitive 1-associated receptor kinase 1
	TRINITY_DN45074_c1_g1	BAK1; brassinosteroid insensitive 1-associated receptor

		kinase 1
	TRINITY_DN43444_c1_g3	BKI1; BRI1 kinase inhibitor 1
	TRINITY_DN36656_c1_g3	BKI1; BRI1 kinase inhibitor 1
	TRINITY_DN32017_c0_g1	BKI1; BRI1 kinase inhibitor 1
	TRINITY_DN39022_c1_g1	BSU1; serine/threonine-protein phosphatase BSU1
	TRINITY_DN37323_c2_g3	BIN2; protein brassinosteroid insensitive 2
	TRINITY_DN47422_c0_g2	BIN2; protein brassinosteroid insensitive 2
	TRINITY_DN45188_c0_g3	BIN2; protein brassinosteroid insensitive 2
	TRINITY_DN36592_c0_g1	BZR1_2; brassinosteroid resistant 1/2
	TRINITY_DN36291_c1_g2	TCH4; xyloglucan:xyloglucosyltransferase TCH4
	TRINITY_DN32798_c2_g1	TCH4; xyloglucan:xyloglucosyltransferase TCH4
	TRINITY_DN36291_c1_g4	TCH4; xyloglucan:xyloglucosyltransferase TCH4
	TRINITY_DN46831_c1_g4	TCH4; xyloglucan:xyloglucosyltransferase TCH4
	TRINITY_DN42269_c0_g1	TCH4; xyloglucan:xyloglucosyltransferase TCH4
	TRINITY_DN32623_c0_g2	TCH4; xyloglucan:xyloglucosyltransferase TCH4
	TRINITY_DN46831_c1_g5	TCH4; xyloglucan:xyloglucosyltransferase TCH4
	TRINITY_DN36291_c1_g1	TCH4; xyloglucan:xyloglucosyltransferase TCH4
	TRINITY_DN36291_c1_g6	TCH4; xyloglucan:xyloglucosyltransferase TCH4
	TRINITY_DN36706_c0_g1	TCH4; xyloglucan:xyloglucosyltransferase TCH4
	TRINITY_DN33419_c1_g3	TCH4; xyloglucan:xyloglucosyltransferase TCH4
	TRINITY_DN38552_c2_g1	TCH4; xyloglucan:xyloglucosyltransferase TCH4
	TRINITY_DN32798_c2_g5	TCH4; xyloglucan:xyloglucosyltransferase TCH4
	TRINITY_DN41569_c0_g2	TCH4; xyloglucan:xyloglucosyltransferase TCH4
	TRINITY_DN36291_c1_g5	TCH4; xyloglucan:xyloglucosyltransferase TCH4
	TRINITY_DN32623_c0_g1	TCH4; xyloglucan:xyloglucosyltransferase TCH4
	TRINITY_DN7380_c0_g1	TCH4; xyloglucan:xyloglucosyltransferase TCH4
	TRINITY_DN32798_c1_g2	TCH4; xyloglucan:xyloglucosyltransferase TCH4
	TRINITY_DN36819_c0_g4	TCH4; xyloglucan:xyloglucosyltransferase TCH4
	TRINITY_DN43726_c2_g1	TCH4; xyloglucan:xyloglucosyltransferase TCH4
	TRINITY_DN46831_c0_g1	TCH4; xyloglucan:xyloglucosyltransferase TCH4
	TRINITY_DN46831_c1_g2	TCH4; xyloglucan:xyloglucosyltransferase TCH4
	TRINITY_DN33826_c1_g1	TCH4; xyloglucan:xyloglucosyltransferase TCH4
	TRINITY_DN46831_c1_g3	TCH4; xyloglucan:xyloglucosyltransferase TCH4
	TRINITY_DN41676_c3_g2	TCH4; xyloglucan:xyloglucosyltransferase TCH4
	TRINITY_DN46831_c1_g1	TCH4; xyloglucan:xyloglucosyltransferase TCH4
	TRINITY_DN37950_c0_g1	TCH4; xyloglucan:xyloglucosyltransferase TCH4
	TRINITY_DN33781_c0_g6	CYCD3; cyclin D3, plant
	TRINITY_DN38013_c1_g1	CYCD3; cyclin D3, plant
	TRINITY_DN34932_c1_g3	CYCD3; cyclin D3, plant
	TRINITY_DN42821_c0_g3	CYCD3; cyclin D3, plant
	TRINITY_DN35958_c1_g1	CYCD3; cyclin D3, plant
	TRINITY_DN37582_c3_g1	CYCD3; cyclin D3, plant
	TRINITY_DN33937_c0_g1	CYCD3; cyclin D3, plant
	TRINITY_DN38903_c5_g4	CYCD3; cyclin D3, plant
	TRINITY_DN44892_c1_g1	CYCD3; cyclin D3, plant
	TRINITY_DN31024_c0_g1	CYCD3; cyclin D3, plant
	TRINITY_DN38880_c1_g2	CYCD3; cyclin D3, plant
	TRINITY_DN45426_c0_g1	CYCD3; cyclin D3, plant
	TRINITY_DN32533_c0_g1	CYCD3; cyclin D3, plant
	TRINITY_DN35958_c1_g3	CYCD3; cyclin D3, plant
**JA**	TRINITY_DN44830_c1_g1	JAR1_4_6; jasmonic acid-amino synthetase

	TRINITY_DN35358_c0_g4	JAR1_4_6; jasmonic acid-amino synthetase
	TRINITY_DN35358_c0_g3	JAR1_4_6; jasmonic acid-amino synthetase
	TRINITY_DN42410_c0_g2	JAZ; jasmonate ZIM domain-containing protein
	TRINITY_DN42410_c1_g1	JAZ; jasmonate ZIM domain-containing protein
	TRINITY_DN41641_c2_g1	JAZ; jasmonate ZIM domain-containing protein
	TRINITY_DN42410_c0_g1	JAZ; jasmonate ZIM domain-containing protein
	TRINITY_DN33533_c0_g1	JAZ; jasmonate ZIM domain-containing protein
	TRINITY_DN42825_c1_g1	JAZ; jasmonate ZIM domain-containing protein
	TRINITY_DN42825_c1_g2	JAZ; jasmonate ZIM domain-containing protein
	TRINITY_DN36623_c0_g1	MYC2; transcription factor MYC2
	TRINITY_DN44455_c0_g1	MYC2; transcription factor MYC2
	TRINITY_DN40925_c1_g2	MYC2; transcription factor MYC2
	TRINITY_DN39736_c1_g1	MYC2; transcription factor MYC2
	TRINITY_DN40921_c0_g2	MYC2; transcription factor MYC2
	TRINITY_DN36014_c1_g1	MYC2; transcription factor MYC2
	TRINITY_DN43604_c1_g9	MYC2; transcription factor MYC2
	TRINITY_DN43019_c1_g6	MYC2; transcription factor MYC2
	TRINITY_DN41995_c1_g6	MYC2; transcription factor MYC2
	TRINITY_DN33677_c0_g1	MYC2; transcription factor MYC2
	TRINITY_DN41995_c1_g3	MYC2; transcription factor MYC2
	TRINITY_DN33828_c0_g7	MYC2; transcription factor MYC2
	TRINITY_DN40925_c1_g1	MYC2; transcription factor MYC2
	TRINITY_DN41160_c0_g2	MYC2; transcription factor MYC2
**SA**	TRINITY_DN28334_c0_g1	PR1; pathogenesis-related protein 1
	TRINITY_DN38097_c0_g1	PR1; pathogenesis-related protein 1
	TRINITY_DN29134_c0_g1	PR1; pathogenesis-related protein 1
	TRINITY_DN32328_c0_g1	PR1; pathogenesis-related protein 1
	TRINITY_DN25826_c0_g1	PR1; pathogenesis-related protein 1
	TRINITY_DN31314_c0_g1	PR1; pathogenesis-related protein 1
	TRINITY_DN40097_c2_g1	PR1; pathogenesis-related protein 1
	TRINITY_DN42854_c0_g2	TGA; transcription factor TGA
	TRINITY_DN47562_c1_g6	TGA; transcription factor TGA
	TRINITY_DN47562_c1_g1	TGA; transcription factor TGA
	TRINITY_DN32428_c4_g2	TGA; transcription factor TGA
	TRINITY_DN41006_c2_g1	TGA; transcription factor TGA
	TRINITY_DN47591_c0_g4	TGA; transcription factor TGA
	TRINITY_DN40679_c0_g1	TGA; transcription factor TGA
	TRINITY_DN33247_c4_g8	TGA; transcription factor TGA
	TRINITY_DN33247_c4_g2	TGA; transcription factor TGA
	TRINITY_DN42049_c0_g3	TGA; transcription factor TGA
	TRINITY_DN47324_c2_g7	NPR1; regulatory protein NPR1
	TRINITY_DN47324_c2_g1	NPR1; regulatory protein NPR1
	TRINITY_DN43702_c1_g2	NPR1; regulatory protein NPR1
	TRINITY_DN41352_c3_g2	NPR1; regulatory protein NPR1

IAA: indole acetic acid, GA:gibberellin, SA:salicylic acid, JA :jasmonic acid, ETH:ethylene, ABA: abscisic acid, BR :brassinosteroid, CTK: cytokinine.

Table S7. Up- and down-regulated DEGs of profile 0 and 7 in female and male flowers.

Hormone	Female		Male	
	Down-regulated	UP-regulated	Down-regulated	UP-regulated
IAA	TRINITY_DN39053_c0_g9<br>TRINITY_DN33167_c0_g1<br>TRINITY_DN45399_c0_g1<br>TRINITY_DN48307_c2_g1<br>TRINITY_DN38814_c1_g2<br>TRINITY_DN48307_c2_g2<br>TRINITY_DN31085_c0_g1<br>TRINITY_DN31433_c0_g1<br>TRINITY_DN45035_c0_g3<br>TRINITY_DN33819_c1_g3<br>TRINITY_DN48237_c6_g1<br>TRINITY_DN48564_c1_g1<br>TRINITY_DN41416_c2_g5<br>TRINITY_DN41416_c2_g1<br>TRINITY_DN41416_c2_g4	TRINITY_DN44119_c0_g1<br>TRINITY_DN40505_c0_g1<br>TRINITY_DN37207_c0_g8	TRINITY_DN33123_c3_g1<br>TRINITY_DN44779_c2_g2	TRINITY_DN45035_c0_g3
CTK	TRINITY_DN33126_c4_g6	TRINITY_DN31066_c0_g1<br>TRINITY_DN44950_c2_g1		TRINITY_DN43499_c4_g1<br>TRINITY_DN33126_c4_g2<br>TRINITY_DN33126_c4_g6
GA	TRINITY_DN38964_c0_g1<br>TRINITY_DN31079_c0_g1<br>TRINITY_DN38732_c5_g1<br>TRINITY_DN39368_c0_g2<br>TRINITY_DN46794_c1_g5<br>TRINITY_DN32735_c0_g1	TRINITY_DN43198_c0_g3<br>TRINITY_DN32445_c0_g1<br>TRINITY_DN29286_c0_g1<br>TRINITY_DN39418_c1_g4		TRINITY_DN43198_c0_g3<br>TRINITY_DN32445_c0_g1

**ABA**	TRINITY_DN46794_c0_g1<br>TRINITY_DN43774_c2_g1<br>TRINITY_DN30413_c0_g1<br>TRINITY_DN39839_c0_g1<br>TRINITY_DN41342_c0_g5<br>TRINITY_DN37658_c0_g3<br>TRINITY_DN31584_c0_g1	TRINITY_DN34170_c2_g4<br>TRINITY_DN41397_c0_g5<br>TRINITY_DN48042_c4_g2<br>TRINITY_DN31174_c0_g1		TRINITY_DN27555_c0_g1<br>TRINITY_DN31755_c0_g1<br>TRINITY_DN48042_c4_g2<br>TRINITY_DN39876_c1_g1
**ETH**	TRINITY_DN37925_c1_g1<br>TRINITY_DN44001_c0_g3<br>TRINITY_DN42852_c1_g2	TRINITY_DN37135_c1_g4	TRINITY_DN44414_c0_g2	TRINITY_DN43293_c1_g1
**BR**	TRINITY_DN34785_c0_g3<br>TRINITY_DN36402_c1_g3<br>TRINITY_DN34785_c0_g2<br>TRINITY_DN45285_c0_g5<br>TRINITY_DN40594_c0_g2<br>TRINITY_DN37394_c1_g8<br>TRINITY_DN38973_c0_g1<br>TRINITY_DN47387_c1_g2<br>TRINITY_DN39121_c0_g2<br>TRINITY_DN47030_c2_g1<br>TRINITY_DN37902_c1_g1<br>TRINITY_DN46222_c0_g3<br>TRINITY_DN32798_c2_g5<br>TRINITY_DN46831_c1_g2<br>TRINITY_DN46831_c1_g3<br>TRINITY_DN37950_	TRINITY_DN36291_c1_g4<br>TRINITY_DN36291_c1_g6<br>TRINITY_DN36706_c0_g1<br>TRINITY_DN33419_c1_g3<br>TRINITY_DN32798_c1_g2<br>TRINITY_DN43726_c2_g1<br>TRINITY_DN46831_c1_g1	TRINITY_DN32453_c0_g2<br>TRINITY_DN36291_c1_g4<br>TRINITY_DN39708_c1_g1<br>TRINITY_DN46311_c3_g3<br>TRINITY_DN39105_c1_g2	TRINITY_DN41569_c0_g2<br>TRINITY_DN46831_c1_g3<br>TRINITY_DN37950_c0_g1

**JA**	c0_g1<br>TRINITY_DN33781_c0_g6<br>TRINITY_DN42821_c0_g3<br>TRINITY_DN35358_c0_g4<br>TRINITY_DN42825_c1_g1<br>TRINITY_DN42410_c0_g2<br>TRINITY_DN42410_c1_g1<br>TRINITY_DN43604_c1_g9	TRINITY_DN33533_c0_g1<br>TRINITY_DN39736_c1_g1	TRINITY_DN43604_c1_g9<br>TRINITY_DN41160_c0_g2	TRINITY_DN41995_c1_g3
**SA**	TRINITY_DN40097_c2_g1<br>TRINITY_DN41006_c2_g1	TRINITY_DN28334_c0_g1<br>TRINITY_DN25826_c0_g1<br>TRINITY_DN32428_c4_g2		TRINITY_DN25826_c0_g1<br>TRINITY_DN47562_c1_g6
